# Soft Multiaxial Strain Mapping Interface with AI-Driven Decoding for Silent Speech in Noise

**DOI:** 10.34133/cbsystems.0536

**Published:** 2026-03-23

**Authors:** Sunguk Hong, Junyoung Yoo, Sung-Min Park

**Affiliations:** ^1^ Department of Mechanical Engineering, Pohang University of Science and Technology (POSTECH), Pohang 37673, South Korea.; ^2^ Department of Creative IT Engineering, Pohang University of Science and Technology (POSTECH), Pohang 37673, South Korea.; ^3^ School of Convergence Science and Technology, Pohang University of Science and Technology (POSTECH), Pohang 37673, South Korea.; ^4^ School of Interdisciplinary Bioscience and Bioengineering, Pohang University of Science and Technology (POSTECH), Pohang 37673, South Korea.; ^5^ Department of Electrical Engineering, Pohang University of Science and Technology (POSTECH), Pohang 37673, South Korea.; ^6^ Institute of Convergence Science, Yonsei University, Seoul 120-749, South Korea.

## Abstract

Silent speech interfaces (SSIs) offer a viable alternative to traditional microphones in capturing clear audio in noisy environments. We propose a reconceptualized SSI that reproduces voice by monitoring continuous multiaxial strain maps induced by throat muscle movements. The system integrates a computer vision-based optical strain (CVOS) sensor with deep learning-based voice reconstruction, enabling clear alphabetic communication under extreme noise conditions. The CVOS sensor—comprising a soft silicone substrate with micromarkers and a tiny camera—achieves high-sensitivity marker detection and captures complex strain patterns with higher scalability and reliability compared to conventional wearable sensors. The inference pipeline of the CVOS-based SSI incorporates physics-based automated baseline calibration and content-adaptive temporal attention, enabling robust analysis of the captured strain patterns. Based on the inference results, a personalized text-to-speech model subsequently reconstructs the speaker’s voice. These algorithmic features ensure robustness under dynamic conditions by employing real-time adaptive signal processing that compensates for inter- and intrasubject anatomical variability. Alphabet-based communication is achieved through the synergy between optimized algorithms and interface design. The performance of the CVOS-based SSI was validated in real-world noisy scenarios, confirming its practical applicability.

## Introduction

Noisy environments hinder effective communication between human beings, which is a crucial component of social life, particularly when clear communication is crucial for the success of an operation, such as in industrial [1,2], military [3,4], and clinical settings [5,6]. In this context, silent speech interfaces (SSIs) have garnered attention as viable alternatives to traditional microphones that struggle to capture sound effectively in extremely noisy environments [7,8]. Traditional SSI systems primarily rely on the measurement of nonverbal biometric signals, such as mechanical motion [9–13], electroencephalography (EEG) [14–16], surface electromyography (sEMG) [17–19], and electrocorticography [20–22], by using wet or invasive sensors and converting these indirect signals into verbal expressions. However, they are usually attached directly to the user’s skin or invasively implanted into their body; this diminishes their usability and substantially restricts their practical application. Thus, next-generation SSI technology must address these challenges to be practically applicable in real-world environments.

To overcome the aforementioned challenges, several innovations have been developed to integrate flexible, high-performance wearable strain sensors within reusable body braces, such as neck chokers and masks, enabling the capture of subtle facial and neck muscle movements [23,24]. The transition of traditional SSI technologies to predominantly wearable forms for sensing strain is expected to enable widespread practical applications and enhance user experience. However, the optimal characteristics of such sensors and the structural design of associated interfaces have not been optimized. Conventional wearable strain sensors measure deformation based on resistance changes induced by stretching of conductive layers. However, their real-world applicability is limited by low reproducibility arising from complex micro- and nanofabrication processes. In addition, these sensors are vulnerable to environmental noise and long-term performance degradation [25–30]. Additionally, and most importantly, because these sensors typically measure strain magnitude only along a single axis, they may fail to capture sufficient information to analyze complex muscle and facial deformation patterns in some cases [31,32], diminishing performance quality and reliability while decoding biometric signals to voice data. These challenges must be addressed by fundamentally improving the design and functionality of wearable strain sensors.

Signal processing algorithms in SSIs transform captured biometric patterns into speech signals, revealing relevant features. Recent research has demonstrated that deep learning-enhanced SSIs can accurately distinguish between a dataset of words [9,23]. Despite these remarkable advancements in deep learning-based signal processing technology for SSI, formidable challenges remain in bridging the gap between theoretical advancements and the practical usability of these devices; innovative approaches are therefore necessary to overcome these limitations. For instance, signal pattern inconsistencies caused by reattachment pose a notable challenge in reusable SSI devices, degrading the performance of deep learning-based inference models. As algorithm tuning or retraining of the inference model upon each attachment of the SSI is impractical, adaptive algorithms are required to autonomously assess the sensor’s alignment status and enable customized inference accordingly to mitigate this issue. Moreover, integration of user voice synthesis technology, which converts decoded biometric patterns to the user’s voice via artificial intelligence (AI)-based text-to-speech (TTS) technology, is essential to develop wearable systems capable of supporting voice-based communication. TTS technology, widely used in over-the-top platforms, is capable of replicating a speaker’s intonation with near-perfect accuracy based on recordings of the speaker’s voice that are less than 10 min in length [33]. Thus, leading-edge signal processing algorithms should be developed by integrating advanced AI technologies to ensure signal integrity in wearable devices under varying attachment conditions and enable accurate voice communication.

**Fig. 1.**
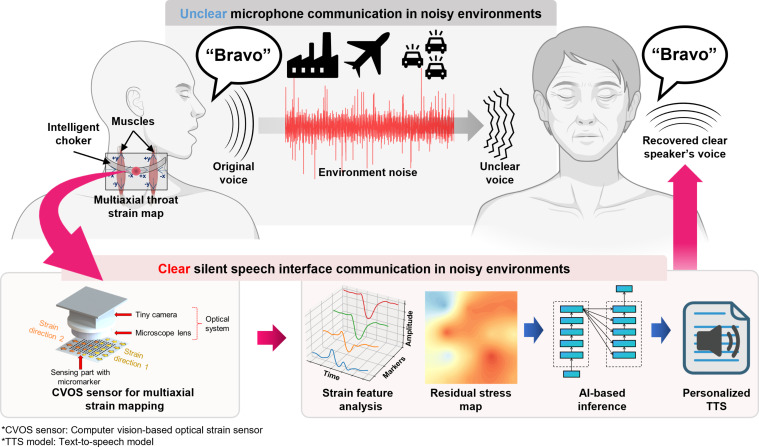
Comprehensive overview of the proposed wearable SSI system, consisting of a reliable multiaxial strain sensor with real-time adaptive speech decoding and reconstruction features.

To address these challenges, we hypothesize that reliable strain sensors capable of monitoring continuous multiaxial throat strain maps can improve SSI performance. In particular, combining such sensing with adaptive AI-based signal processing within a spelling alphabet communication framework is expected to enhance both usability and robustness. Here, we introduce a reliable multiaxial strain sensor-based SSI with adaptive speech decoding and regeneration, which integrate 3 key technical concepts (Fig. 1). First, complex throat muscle movements are measured using a computer vision-based optical strain (CVOS) sensor [31]. The CVOS sensor overcomes the durability and reproducibility issues of conventional wearable strain sensors—often compromised by oxidation and degradation of conductive layers—by combining optical strain sensors with edge computer vision technology. The CVOS sensor accurately captures a multiaxial strain map enabling the extensive acquisition of vector information, including not only the magnitude but also the direction of strain on the attached surfaces. Therefore, when integrated with AI, it allows for precise analysis of not only voice but also various other types of information, making it a scalable solution adaptable to diverse applications. As demonstrated in this study, the interface with the CVOS sensor is reusable, reproducible, and reliable, thus satisfying the requirements of real-world SSI applications. Second, the proposed interface is designed to recognize the North Atlantic Treaty Organization (NATO) phonetic alphabet, which has been in use since the 1960s to minimize misinterpretation of spoken words in noisy wireless communications, particularly in military and industrial environments [34]. For example, the commonly used communication phrase “Loud and Clear” is converted to “Lima Charlie” by using the NATO phonetic alphabet. Combining the NATO phonetic alphabet with SSI enables diverse expressions to be communicated using a limited set of words, broadening the potential applications of SSIs. Finally, the multiaxial strain map generated during the pronunciation of the phonetic alphabet, captured by the throat-mounted CVOS sensor, is decoded using a real-time adaptive deep learning-based signal processing algorithm. The proposed vocal classification model is integrated with an adaptive algorithm that compensates for signal waveform differences induced by varying wearing conditions and individual differences, ensuring broad generalizability across users. Further, the classified signals are reconstructed in the speaker’s actual voice using a TTS model, thereby enhancing communication convenience for listeners. In particular, personalized models tailored to an individual’s unique physical and pronunciation characteristics are created based on integrated AI technologies trained on recordings of 10 min or less, enabling a highly customizable and efficient SSI system. Recognition performance tests conducted in environments with irregular noise levels (~90 dB) demonstrated the remarkable capability of the proposed system for effective communication in extremely noisy environments. The test results indicate that the proposed next-generation SSI, featuring a meticulously designed system architecture, leading-edge signal processing algorithms, and specialization in phonetic alphabet communication, exhibits a wide scope of application. It overcomes the fundamental limitations of existing systems and is expected to be comprehensively adopted in real-world situations that demand high reliability.

## Materials and Methods

### CVOS sensor for wearable SSI

#### Sensing part

White Ecoflex (Ecoflex 0030, Smooth-On Inc., Macungie, PA, USA) prepolymer was prepared by mixing the base, curing agent, and white silicone pigment (Silc Pig, Smooth-On Inc., Macungie, PA, USA) in a weight ratio of 1:1:0.01. The prepolymer was spin-coated onto a large glass slide (76 mm × 52 mm) at 1,000 rpm for 10 s and cured at 60 °C for over 6 h. A CO_2_ laser-marking machine (CM30D, Lasers Co. Ltd., Republic of Korea) was used to create microholes on the white Ecoflex film along a programmed scanning pattern. The laser parameters were set to 24 W power, 20 mm/s scanning speed, and 4 cycles. The micromarkers were produced by laser burning, which generated impurities that interfered with micromarker detection. To remove these impurities, the surface of the white Ecoflex film was cleaned by repeatedly applying and peeling Scotch tape. Black Ecoflex prepolymer was prepared similarly by mixing the base, curing agent, and black silicone pigment at the same weight ratio of 1:1:0.01. The black Ecoflex prepolymer was deposited onto the white Ecoflex film with microholes via spin coating at 1,000 rpm for 10 s, followed by curing at 60 °C for over 6 h. During this process, the black Ecoflex prepolymer fills the microholes of the white Ecoflex film, forming black micromarkers. The laser-marking machine was then used to cut the edge of the sensing part at 24 W, 20 mm/s, and 2 cycles. The total thickness of the sensing part was 200 μm. The overall sensing part fabrication process is shown in Fig. S1.

#### Optical system

The optical system of the CVOS sensor comprised a tiny camera (Raspberry Pi Camera Module 3, Raspberry Pi, United Kingdom/size: 11 mm × 11 mm × 6.4 mm), compact microscope lens (iMicro C, Shanghai Qingying E&T LLC, China/size: ø 12.75 mm × 3.5 mm), and light-emitting diode (LED) light source (LUW/G/B30243, HSUKWANG, China). The tiny camera was connected to an embedded board (Raspberry Pi 5, Raspberry Pi Foundation, United Kingdom) by using a cable. The LED light source was connected to the embedded board with a bias voltage of 3 V.

### Manufacture of neck choker and system integration

The neck choker was fabricated using 1.5-mm-thick neoprene fabric, a 2-mm-thick elastic band, hook-and-loop fasteners (Velcro), open-gate clips, and buckles. The CVOS sensor was attached to a 20-mm-wide strip of neoprene fabric. The combination of the elastic band, Velcro, and open-gate clips allows the length of the choker to be adjusted to fit the user’s body size. The buckle, located at the back of the neck, facilitates easy attachment and removal (Fig. S2).

### Real-time multiaxial strain analysis algorithm of the CVOS sensor

#### Micromarker detection process

First, contrast-limited adaptive histogram equalization was applied to the original image to enhance global contrast and improve the edge definition of the micromarkers by redistributing contrast based on the pixel intensity histogram. Second, unsharp masking was used to sharpen the edges of the micromarkers, followed by the application of a Gaussian blur filter to reduce image noise. The image was then convolved with a sharpening filter:

$$\text{Sharpen filter}=\left[\begin{array}{ccc} -1 & -1 & -1\\ -1 & 11 & -1\\ -1 & -1 & -1 \end{array}\right]$$ (1)

Third, the black boundary caused by misalignment between the optical system, commercial lens, and camera was removed, as it introduced noise during the bounding box extraction process. Fourth, the image was binarized using Otsu’s method, effectively distinguishing the micromarkers from the Ecoflex substrate by assigning distinct colors to them. Finally, the bounding boxes of the micromarkers were extracted from the binarized image. Bounding boxes that were too small or too large were excluded from extraction.

#### Deciding the initial MOIs

In the no-strain state, the Euclidean distance between the center coordinates of the image and each micromarker was calculated using the following equation:

$$\text{Euclidean distance}=\sqrt{{\left(x_{c}-x_{m}\right)}^{2}+{\left(y_{c}-y_{m}\right)}^{2}}$$ (2)

where $\left(x_{c},\;y_{c}\right)\;$ represents the center coordinates of the image and $\left(x_{m},\;y_{m}\right)\;$ represents the center coordinates of each micromarker in Euclidean space. The 16 micromarkers with the smallest Euclidean distances were selected and assigned unique IDs. These selected micromarkers were referred to as markers of interest (MOIs), and their initial coordinates were recorded.

#### Generation of the multiaxial strain map

First, corresponding points between the initial and current positions of the micromarkers were identified using the fast library for approximate nearest-neighbors matcher algorithm. Next, a homography matrix was computed using the RANdom SAmple Consensus method based on the matched points. Third, maps were initialized as empty arrays, and computation coordinates were defined according to a specified grid size. Finally, the grid points were transformed using the homography matrix, and both the strain amplitude map and the strain direction map were determined by comparing the transformed grid points with the original ones. The strain amplitude map was calculated as the Euclidean distance between the transformed and original points, while the strain direction map was derived from the inverse tangent of the directional changes. By integrating these components, the comprehensive multiaxial strain map was finally constructed.

#### Initial residual stress map

First, the average coordinates for the *x* and *y* positions of the captured micromarkers were computed to identify the geometric center of the micromarkers. Second, the base points were adjusted by aligning them with the centroid of the designed micromarkers through translation, thereby ensuring spatial consistency between the designed and captured micromarkers. Finally, the Euclidean distance between the adjusted positions of the designed micromarkers and the corresponding captured micromarkers was calculated to quantify positional deviations, forming an initial residual stress map.

### Real-time AI-based signal processing algorithm

#### Transformer-based classifier

Fig. S3 and Tables S1 and S2 show model structure and hyperparameters. An AI-based multiaxial strain pattern classification model was devised by incorporating a transformer architecture and a convolutional neural network (CNN), and it was optimized using PyTorch. During the data preprocessing stage, the input data were normalized and batched to suit the training process, and shuffling and data augmentation were applied to enhance training diversity. The model architecture consists of an input layer, a CNN-based feature extraction layer, a transformer layer for time-series data, and fully connected layers. Nonlinear activation functions (rectified linear unit) were employed to improve the learning of complex patterns. In the fully connected layer, multiaxial strain pattern information and initial residual stress map information were combined. The Adam optimizer was used for optimization, and cross-entropy was adopted as the loss function to optimize the model for classification tasks. Training was performed over a specified number of epochs, incorporating early termination and model checkpointing to prevent overfitting and ensure optimal performance. Finally, the performance of the model was evaluated on a test dataset, with accuracy and loss metrics used to assess generalization performance. The model implemented using PyTorch was optimized for deployment by converting it to the Open Neural Network Exchange format, which enables compatibility across various platforms and frameworks.

#### Classifier model lightweighting

Table S1 shows model structure and hyperparameters. The knowledge distillation approach was applied to reduce model complexity and improve efficiency while maintaining performance. This approach transfers knowledge from a pretrained large model (Teacher) to a smaller one (Student) without any loss of validity. The Student model was trained using both hard loss and soft loss: Hard loss ensures that the Student matches the Teacher model’s predictions, while soft loss helps the Student learn from the Teacher’s probability.

#### TTS model

The word information inferred from the client (user wearing the SSI device) is transmitted to a server (other user), where the corresponding word is retrieved from a predefined list, synthesized into audio, and delivered to the user via an audio stream. The TTS implementation is based on the open-source project, piper-tts, with a TTS model converted to the Open Neural Network Exchange format deployed on the server.

#### Voice activity detection

The voice activity detection algorithm implements an energy-based detection mechanism to identify segments of a strain signal with pronounced activity. The algorithm computes the energy of the 200 most recent samples by using a convolution operation with a square window of a specified length. If the energy surpasses a defined threshold, then a detection event is registered, and the start index is set. When the energy falls below the threshold, marking the end of the activity, the end index is set, and the segment’s length is validated. The detected voice samples are subsequently converted into meaningful audio signals by using the proposed transformer-based classifier.

### Setup for the performance test of the CVOS sensor

The mechanical properties of the CVOS sensor were evaluated using a universal testing machine (UNITEST M1, TEST ONE Co. Ltd., Republic of Korea). A 3-dimensionally printed mount was designed to hold a small camera, a compact microscope lens, and a LED light source, all integrated for attachment to the testing machine. During the test, an embedded board facilitated real-time image processing and provided power to the LED light source.

### Setup for data acquisition

This experiment was approved by the institutional review board of POSTECH (PIRB-2024-E016). The experiments involving human subjects were performed with the full consent of the volunteers, including the publication of identifiable images. All participants provided written informed consent. The users wore a wearable SSI around the neck, ensuring that the CVOS sensor was positioned below the laryngeal prominence, while pronouncing the NATO phonetic alphabet. Although the placement and tightness of the wearable SSI may vary slightly among users, no strict guidelines were provided to facilitate realistic data collection. The participants consisted of healthy young adults aged 23 to 32 years, including both male and female subjects, with no voice-related disorders (the “old user” group; see Table S3). This demographic range was intentionally selected to accommodate relevant anatomical considerations of the laryngeal region and to support the generalizability of the reported results. To introduce informative variations into the dataset, samples were collected on different dates with varying word-reading speeds and SSI attachment conditions. All participants produced target words while seated. Throughout the extensive data acquisition sessions, no strict guideline was imposed on the SSI device in terms of vertical placement or wearing tightness. Instead, participants were instructed to wear the choker comfortably around the neck at an approximate midcervical height, emulating natural daily-use variability rather than enforcing rigid positioning constraints.

### Training data preparation

The training dataset consists of a total of 5,186 samples collected from 6 participants (the “old user” group; see Table S3), covering all 26 NATO phonetic alphabet words. Each data file contains multiaxial strain time-series signals corresponding to a single spoken alphabet. Although the duration varies slightly depending on the alphabet, each sequence is approximately 4 s on average. To prevent data imbalance, we ensured that each alphabet class contained at least 180 samples, resulting in a uniformly distributed dataset across all 26 categories.

### Data preprocessing and augmentation strategy

#### Data preprocessing

Before feeding the signals into the model, we applied a series of preprocessing steps to standardize the input data and mitigate sensor noise.

1. Noise smoothing: To reduce high-frequency fluctuations inherent in sensor data, a moving average filter with a window size of 5 was applied to the raw time-series signals.
2. Temporal standardization (padding): The valid signal length was defined as ranging between 100 and 220 time steps. Signals shorter than the maximum allowable sequence duration were symmetrically zero-padded (center padding) to produce a unified input length of 220 time steps prior to augmentation.
3. *z*-score normalization: To ensure stable convergence during training, all signals (*x*) were normalized using global statistics computed from the training set:
  $$x_{\mathrm{norm}}=\frac{x-\mu_{\text{train}}}{\sigma_{\text{train}}}$$ (3)
  where *μ*_train_ and *σ*_train_ denote the mean and SD of the entire training corpus, respectively.

#### Data augmentation

To improve the model’s generalization capability and robustness against temporal and intensity variations, we employed a stochastic data augmentation strategy during the training phase. The following 3 transformations were applied sequentially in the data loader.

1. Random time warping (local dilation): To simulate variations in the speed of motion, we randomly selected 20 time indices within the active signal range (indices 20 to 200) and duplicated the values at these steps. This process locally stretches the temporal axis, introducing nonlinear time warping effects.
2. Random temporal cropping (shift invariance): From the padded and warped sequence (which exceeds the target length), a fixed-size window (200 time steps) was randomly cropped. This technique allows the model to learn features invariant to the start timing of the action (i.e., temporal shift invariance).

$$x_{\text{crop}}=x_{\text{warped}}\;\left[t_{\text{start}}:t_{\text{start}}+200\right]$$ (4)

3. Random amplitude scaling (multiplicative noise): To simulate sensor sensitivity fluctuations or varying signal intensities, we applied multiplicative noise. The cropped signal was scaled by a factor *α* sampled from a uniform distribution:

$$x_{\mathrm{aug}}=x_{\text{crop}}\cdot \alpha ,\text{where}\;\alpha ∼u\left(0.9,1.1\right)$$ (5)

#### Training/test set split

To ensure a robust and unbiased evaluation of the proposed model, we employed a stratified 5-fold cross-validation scheme under a subject-independent setting. Instead of splitting the entire dataset globally, we applied the partitioning procedure separately to each class while simultaneously enforcing subject-level separation. Specifically, for every class, samples were randomly shuffled and divided into 5 disjoint subsets, ensuring that no data originating from the same individual appeared in both the training and testing folds. In each iteration, 4 subsets (80%) were aggregated to form the training set, whereas the remaining 1 subset (20%) was reserved for testing. This combined class-wise and subject-independent splitting strategy guarantees both a consistent label distribution across folds and prevention of data leakage arising from intrasubject overlap. Consequently, we conducted 5 independent experiments and reported the average performance metrics. The batch size was set to 256 during training to stabilize gradient updates and 1 during testing to simulate deployment-level single-sample inference.

### Setup for the signal baseline measurement of the CVOS sensor and speech recognition test according to sound level

The signal baseline of the CVOS sensor was measured by gradually increasing the sound level using a surround speaker in an enclosed space. Speech recognition test was conducted in the same environment with 90-dB white noise, while specific words were pronounced, allowing for the measurement of the CVOS sensor’s signals. The sound level was evaluated and measured using a portable sound-level meter (DT-95, Shenzhen Everbest Machinery Industry Co. Ltd., China).

### Setup for the experimental demonstration

The speech recognition performance of the wearable SSI with the CVOS sensor was tested while wearing it around the neck and firing a gas blowback rifle (GBBR; AKX, Tokyo MARUI, Japan) to make sound noise and mechanical vibration. During the test, the signals and inference results from the wearable SSI were transmitted in real time to a monitoring laptop via socket communication. The real-time noise levels were measured using the portable sound-level meter.

## Results

### CVOS sensor placed on the throat for highly sensitive and reliable recognition of the multiaxial strain map

Wearable strain sensors, a key component of wearable SSI devices, suffer from marked limitations that hinder their practical application. Unintended deformation of the wearable system during use may damage the conductive layers, compromising their long-term performance. Moreover, conventional wearable strain sensors manufactured via complex and specialized micro-/nanofabrication processes exhibit substantial performance variations due to discrepancies between manufacturing samples. These variations can lead to unreliable data for advanced SSI technologies, posing substantial challenges in inference model reliability. To address these issues, we devised a scalable solution that integrates redesigned sensing mechanisms with universally manufacturable micromarkers, ensuring greater reliability and reproducibility across devices.

**Fig. 2.**
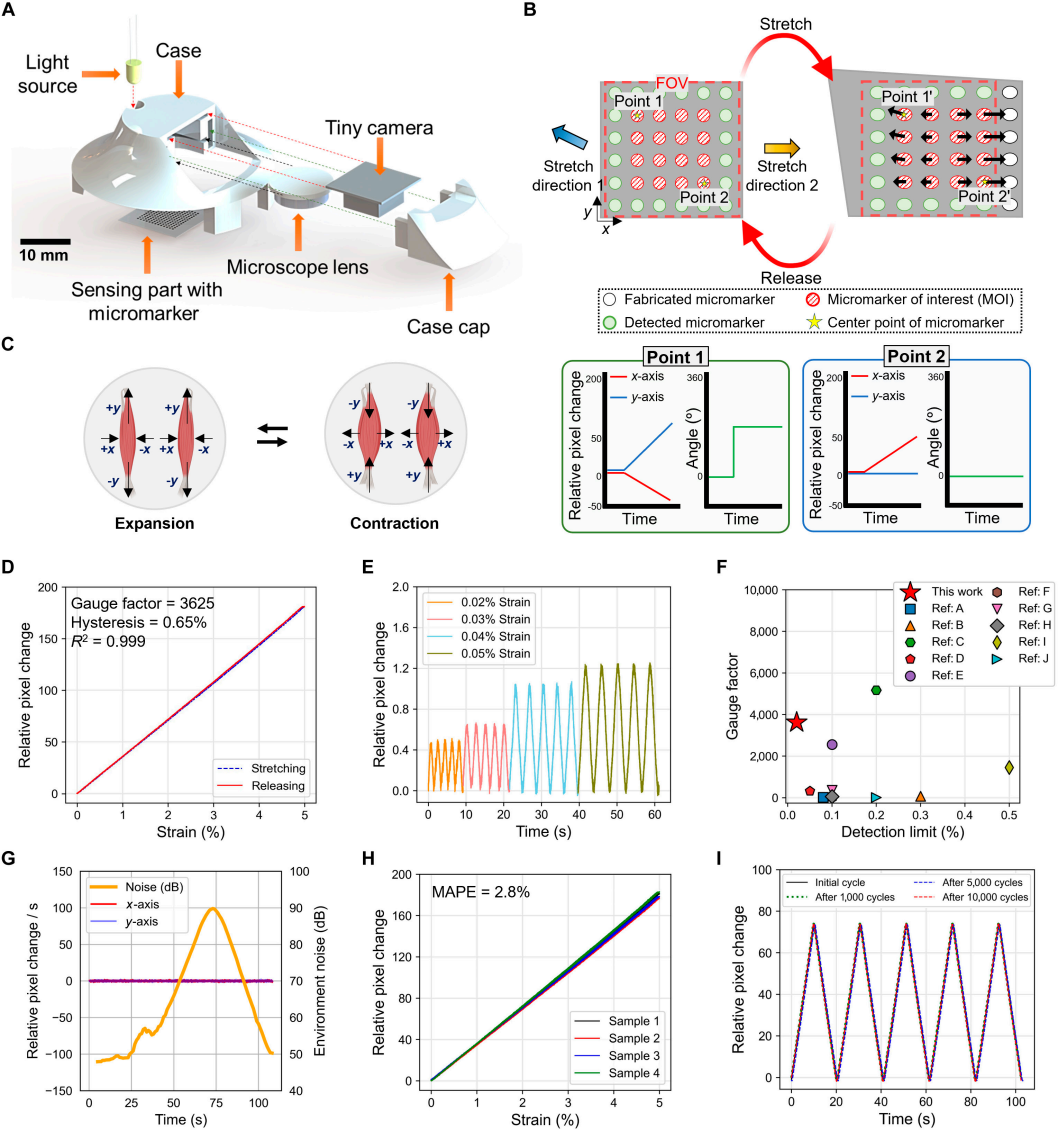
Design, mechanism, and mechanical characteristics of the CVOS sensor. (A) Design and configuration of the CVOS sensor. (B) Multiaxial strain map detection mechanism and corresponding micromarker movement patterns in multiaxial strain directions. (C) Multiaxial strain map during the expansion and contraction phases of throat muscle movement. (D) Relative pixel change during loading–unloading cycles at 0% to 5% strain. (E) Relative pixel change corresponding to 0.02%, 0.03%, 0.04%, 0.04%, and 0.05% cyclic strains. (F) Comparison of the gauge factor and detection limit of the strain sensors with those of previously reported wearable strain sensors. (G) Baseline signal of the CVOS sensor under external noise levels of 50 to 90 dB. (H) Sensor-to-sensor uniformity for 4 samples at 0% to 5% strain. (I) Comparison of test results obtained with the initial number of cycles and after 1,000, 5,000, and 10,000 cycles.

Capturing the complex micromovements of throat muscles accurately and developing an effective signal-to-speech conversion algorithm require a highly sensitive and reliable wearable strain sensor with a multiarray structure capable of simultaneously capturing the magnitude and direction of multiaxial strain. In contrast, most previous studies have focused on single-axis capturing. Fig. 2A illustrates the CVOS sensor, comprising a sensing component constructed with micromarkers placed on an Ecoflex film (~100 μm thick) and an optical component constructed using a microcamera and a microscope lens that capture positional changes in the micromarkers, with each component sized to minimize any impact on the overall flexibility of the device. The opaque white substrate of the sensing component and the black circular micromarkers maximize the recognition rate of the optical system by implementing a high-contrast color scheme and minimize the resources required for image processing by preventing unnecessary light reflection and penetration. The optical system is adjusted to capture the micromarker positions from a small distance (1 to 3 mm) at a resolution of 320 × 240 pixels to enable high-speed image processing. Fig. 2B illustrates the multiaxial strain map detection mechanism of the CVOS sensor. Within the field of view (FOV; red dashed box), the CVOS sensor designates the micromarkers (green circles) located near the center as MOIs (red circles) and tracks their coordinates to measure both the magnitude and direction of the deformation of each MOI. In this process, the magnitude (i.e., pixel change) is computed as the Euclidean distance between the initial and displaced coordinates, while the direction of deformation (i.e., angle) is derived from the arctangent of the displacement vector formed by these coordinates. By analyzing these results, the system generates a multiaxial strain map that includes the magnitude and direction data of local deformations (Movie S1).

Fig. 2C illustrates the multiaxial strain map during throat muscle motion, highlighting the importance of integrating multiaxial strain mapping sensors, such as CVOS sensors, within wearable SSI devices. Biomechanical models of the vocal folds show that phonation involves complex, spatially varying deformations with asymmetric and nonplanar motion, reinforcing the need to capture multiaxial strain patterns rather than a single scalar displacement [35,36]. In the expansion phase, the throat muscle contracts along the *x*-axis and elongates along the *y*-axis, whereas during the contraction phase, the throat muscle elongates along the *x*-axis and contracts along the *y*-axis. These complex muscle movements form the multiaxial strain map, capturing the local features of the throat skin.

The performance of the CVOS sensor is evaluated based on variations in the relative pixel change of the MOIs, which is defined as follows:

$$\text{Relative pixel change}=\frac{∆\mathrm{MOI}}{\mathrm{MO}I_{0}}$$ (6)

where MOI₀ represents the initial coordinates of the MOI’s center and ΔMOI denotes the difference between the initial and current coordinates of the MOI center. The performance of the CVOS sensor depends on factors such as the distance between the sensing component and the lens, the size of the sensing component, and the pitch of the pattern [31]. In this study, these factors were fine-tuned to capture neck muscle movements accurately. The high gauge factor (3,625), low hysteresis (<0.65%), and high linearity (>0.99) at the working range (*ε* = 0% to 5%) depicted in Fig. 2D highlight the superior performance of the CVOS sensor in capturing biomechanical deformations. As illustrated in Fig. 2E, the CVOS sensor obtained stable signals corresponding to strains of 0.02% to 0.05%. The ultralow detection limit of 0.02%, which is close to the detection limits of the most recently developed wearable strain sensors and is a critical factor in capturing ultrafine biomechanical movements of throat muscles, combined with the unique and highly sensitive multiaxial strain mapping capabilities of the CVOS sensor, enables the measurement of highly complex deformation patterns (Fig. 2F and Table S4). Fig. 2G illustrates the changes in the baseline of the CVOS sensor corresponding to environmental noise of 50 to 90 dB (Movie S2). The CVOS sensor exhibits an extremely sensitive response to minute direct movements of the skin on the neck. However, it does not depend on environmental noise levels exceeding 50 dB. The combination of a sensor with minimal influence of unnecessary environmental noise and SSI enhances the robustness of the signal processing algorithm, thereby improving the reliability of SSI. Fig. 2H illustrates the minimal performance differences between the 4 CVOS sensor samples, indicating high uniformity across test specimens with a mean absolute percentage error (MAPE) of 2.8%. MAPE is defined as follows:

$$\text{MAPE}=\frac{100\%}{n}\;\sum_{t=1}^{n}\left|1-\frac{{\mathrm{AS}}_{t}}{S_{t}}\right|$$ (7)

where n denotes the number of data points, $S_{t}$ denotes the response of a sensor, and ${\mathrm{AS}}_{t}$ denotes the response of another sensor. The high uniformity is attributed to the scalable fabrication methodology of the CVOS sensor using precise laser processing and commercial components, without the need for complex nano-/microfabrication processes. Fig. 2I depicts the test results after 1,000, 5,000, and 10,000 loading–unloading cycles, compared to the result corresponding to the initial number of cycles corresponding to a strain range of 0% to 2%. The nearly identical curves corresponding to the different numbers of cycles demonstrate the high reliability and durability of the CVOS sensor. Its mechanical stability and reusability guarantee its practical applicability (Movie S3). Overall, its ultralow detection limit, high sensitivity, high linearity, uniformity across sensors, and high durability provide distinct competitive advantages, enabling the development of practical SSI applications compared to sensors based on other sensing mechanisms and materials.

### Correlation of initial residual stress maps and strain patterns

**Fig. 3.**
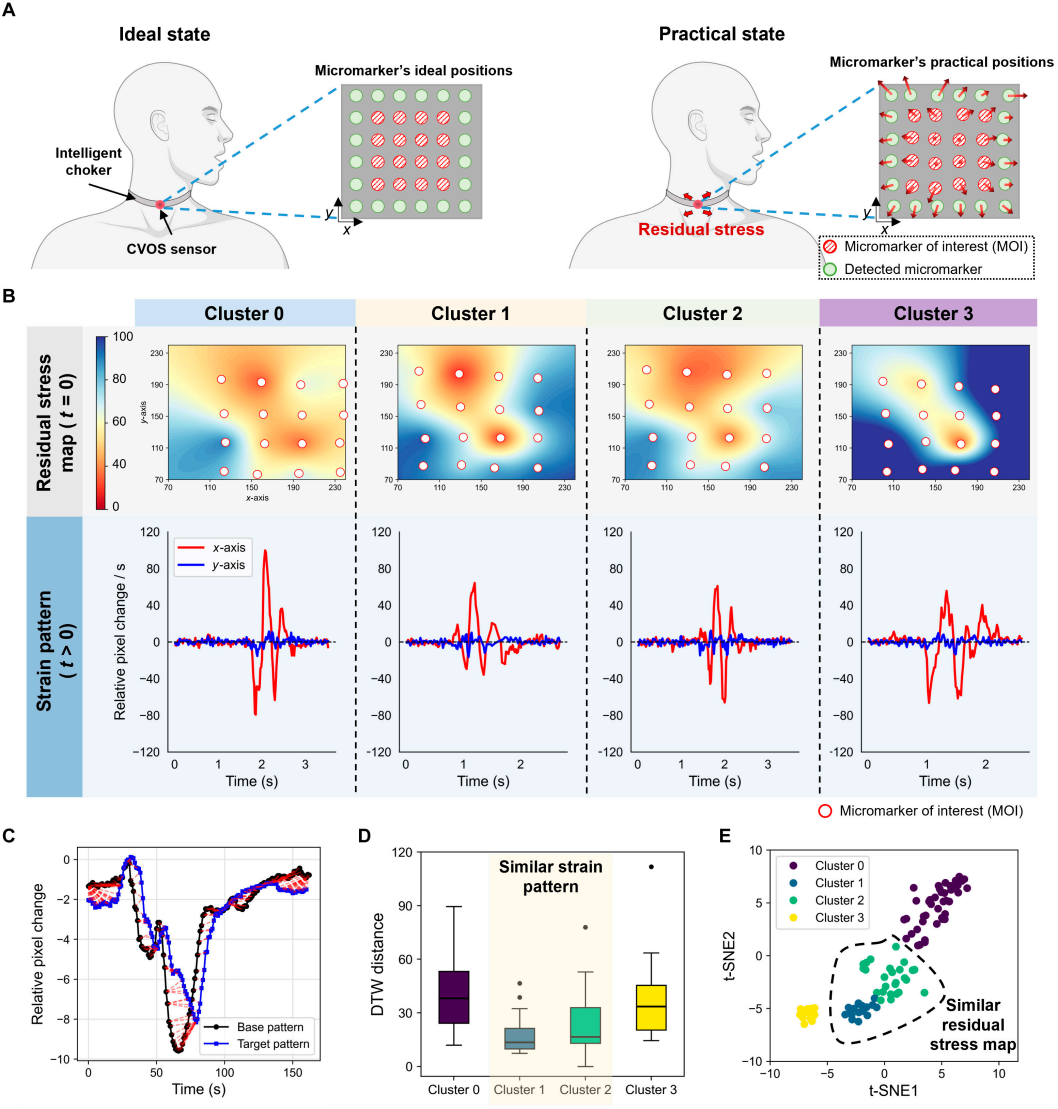
Strain patterns with respect to initial residual stress maps. (A) Positional changes in micromarkers in the CVOS sensor in the absence (ideal state) or presence (practical state) of initial residual stress maps. (B) Strain patterns with respect to residual stress map clustering, revealing the relationship between the initial residual stress map and the strain pattern for each word. (C) DTW-based method enables similarity calculations between the base and target patterns even in the presence of distortions. (D) Box plot of DTW distance, demonstrating the similarity between strain signal patterns corresponding to Clusters 1 and 2, with the base pattern of DTW located in Cluster 2. (E) The t-SNE visualization of clustering results for initial residual stress maps enables the analysis of similar initial residual stress map patterns.

In practical scenarios, wearable systems record varying strain signal patterns corresponding to the same original vocal signal owing to initial residual stress map (Fig. 3A). This map represents the spatial distribution of preexisting geometric deformation imposed on the sensor–skin interface prior to intentional speech-related muscle activation. The initial residual stress map can vary from trial to trial depending on factors such as the device–skin contact condition, tightening force, and attachment position, consequently leading to differences in the measured strain patterns even for identical utterances. Fig. 3B presents representative images of clustered initial residual stress maps captured by the proposed wearable SSI device during the recognition of the word “Oscar” and the corresponding strain patterns across cluster groups, depicting the relationship between initial residual stress maps and strain patterns for the same word. The initial residual stress map is generated based on the differences between the initial and current positions of the micromarkers. This relationship was numerically verified in terms of the dynamic time warping (DTW) distance based on the similarity between the base pattern (located within Cluster 2) and each strain pattern (Fig. 3C). DTW enables similarity calculations between base and target patterns even in the presence of distortions or time delays in time-series data, with greater similarity corresponding to lower DTW distances (Fig. 3D). In this study, the DTW distance analysis demonstrated that the strain patterns in Clusters 1 and 2 exhibited a high degree of similarity. Fig. 3E depicts a 2-dimensional latent space visualization of the initial residual stress maps based on t-distributed stochastic neighbor embedding (t-SNE), indicating that Clusters 1 and 2 formed similar groups in adjacent positions within the latent space. In conclusion, the initial residual stress map was observed to be highly correlated with the strain pattern, emphasizing the necessity of measuring initial residual stress maps using multiarray sensors, such as the CVOS sensor, and developing signal processing algorithms that account for the diversity of initial residual stress maps to achieve accurate pattern recognition using wearable SSI.

### Integration of signal processing algorithm with deep learning models for speech recognition and regeneration

**Fig. 4.**
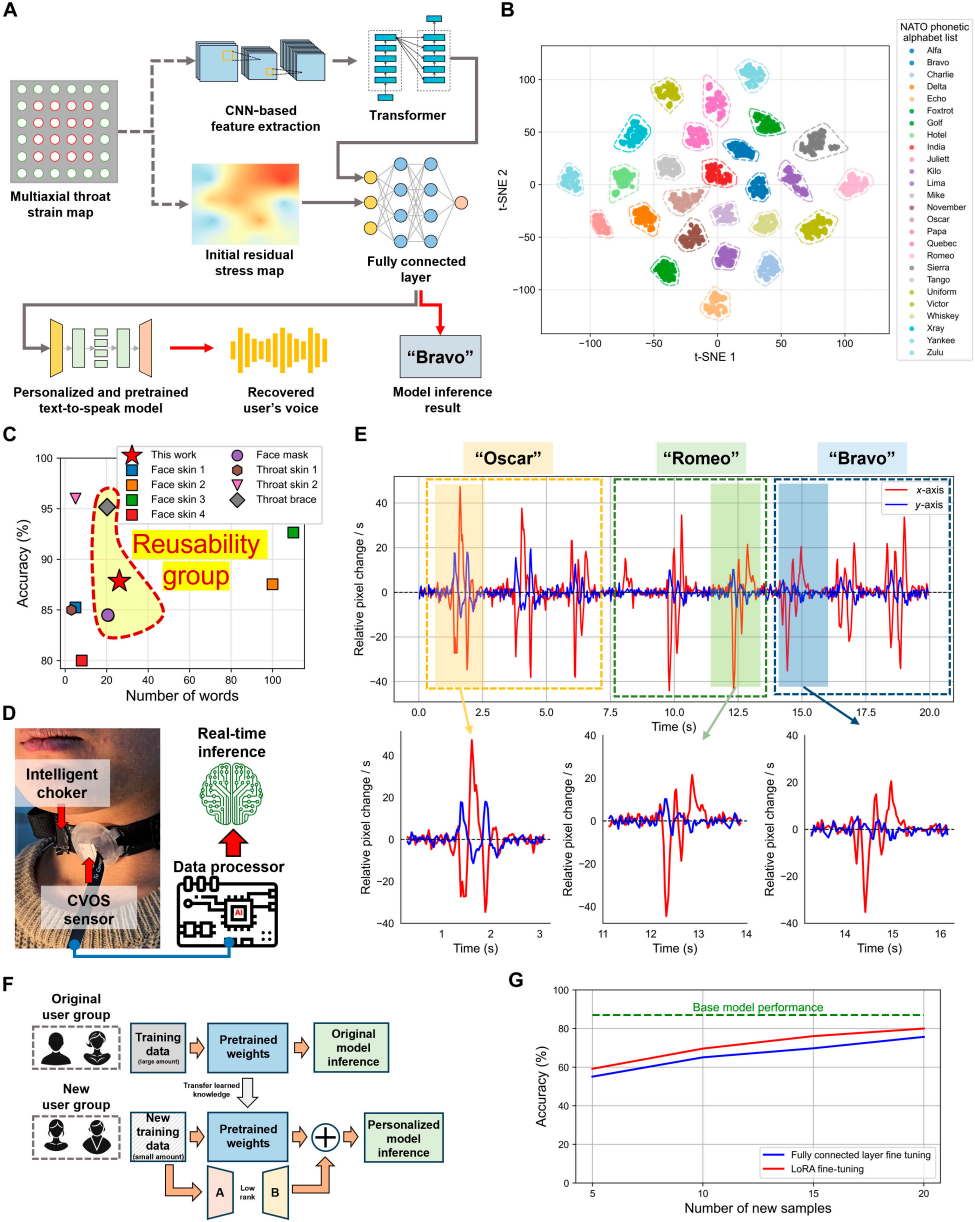
Overall algorithm structure and real-time inference performance. (A) Overall signal processing flowchart. (B) t-SNE visualizations depicting model classification performance for NATO phonetic alphabet recognition. (C) Performance comparison of SSI classification models, highlighting reusability in the SSI group. (D) Experimental environment for evaluating real-time word recognition using the CVOS-based SSI. (E) Real-time inference performance of SSI integrated with the proposed AI model. (F) Flowchart depicting the generalization process based on LoRA for the model. (G) Evaluation of the model’s generalization performance—accuracies obtained based on training from fully connected layer fine-tuning and LoRA fine-tuning using varying amounts of new user samples are compared.

Fig. 4A presents a flowchart of the signal processing algorithm. Recently proposed signal processing algorithms for strain sensor-based SSI systems typically follow a deep learning structure of “strain feature extraction + neural network classifier”. However, as indicated by the aforedescribed results, practical algorithms must include a flow that accounts for the initial alignment state of the sensors as an automatic calibration process. In addition, the data output after classification should be converted into speech using TTS technology. Therefore, we adopted the algorithm flow of “strain feature extraction + initial sensor alignment state + transformer classifier + TTS”, supporting a highly reliable and practical SSI system. The training dataset comprises multiaxial strain map data obtained using the throat-mounted CVOS sensor, along with initial alignment state maps containing initial residual stress information, enabling the development of a robust classifier that performs reliably corresponding to various attachment states and individual variations through intelligent adjustment of measurement deviations. Previous studies on SSI systems have primarily adopted CNN- and temporal convolutional network-based classifiers for articulatory pattern recognition. Convolutional networks provide strong local feature extraction capabilities. However, their reliance on fixed receptive fields limits their ability to capture multiscale temporal variability and nonlocal coarticulatory interactions inherent in silent speech signals [37,38]. In particular, CNN-based architecture tends to emphasize localized strain responses without adequately modeling nonlocal coarticulatory coupling, temporal drift, and subject-dependent biomechanical variability. To address these constraints, we employed a hybrid CNN–Transformer architecture, in which the CNN component captures fine-grained spatial deformation cues, whereas the transformer module performs attention-based temporal reweighting and global alignment across deformation trajectories. This design allows the model to compensate for temporal instability and biomechanical variability that are not well represented by convolution alone. The backbone structure and hyperparameters of the transformer-based classification network are detailed in Fig. S3 and Tables S1 and S2. Although the patterns analyzed by the classifier can be represented in text form, we used TTS to recreate the user’s actual voice. The TTS model can be personalized based on training on user vocal recordings that are 10 min or less in length.

Wearable SSI devices, typically integrated with body braces (e.g., neck choker), can experience signal attenuation owing to the thickness and material properties of the brace itself. This may result in distortions in strain patterns, thereby blurring the distinction between similar word patterns (e.g., “book” and “look”) and increasing the likelihood of classification errors. To avoid this problem, we adapted the NATO phonetic alphabet system, an internationally recognized standard used to convey letters clearly, particularly in radio, telephone, and other voice-based communications. In this standard, each letter corresponds to a distinct word to avoid miscommunication caused by similar-sounding letters. For instance, “A” is represented by “Alpha”, “B” by “Bravo”, and “C” by “Charlie” (Table S5). This system is widely used in communication in the industry; military, aviation, and maritime sectors; emergency services; and civilian scenarios wherein clear communication is critical. Fig. 4B visualizes the classification performance of the proposed model for the NATO phonetic alphabet using t-SNE, demonstrating the model clusters for each letter in a low-dimensional space. The clusters for the different letters are visually distinct from one another, indicating the high accuracy and structural robustness of the model corresponding to the NATO phonetic alphabet (Figs. S4 to S6).

The proposed classification network achieves an accuracy of 85.8% for the 26 NATO phonetic alphabet words, as shown in the confusion matrix in Fig. S7 and the submodel performance and architecture (temporal convolutional network, CNN, and Hybrid model with number of channels and the multiaxial strain map) in Fig. S8 and Tables S6 and S7. The overall performance of the developed classifier positions itself as a viable option for SSI, particularly for the reusability group designed for integration with body braces (Fig. 4C and Table S8). Additionally, for edge computing, we applied knowledge distillation using the Teacher–Student approach to minimize the size of the classification network (12.4 MB → 3.6 MB), thereby minimizing processing speed (0.018 s → 0.003 s and Fig. S9) while maintaining model performance (85.8% → 82%). Figs. 4D and E and Movie S4 present the real-time inference results of the SSI integrated with the proposed AI model. When the user wearing the SSI articulates the words “Oscar,” “Romeo,” and “Bravo,” the AI-based real-time data processing algorithm is shown to analyze the input successfully and infer the spoken words with high accuracy (including 1 instance of misclassification in the provided material). In the same experiment, repeated 2 weeks later, despite slight variations in the initial residual stress map due to the reattachment of the SSI previously used by the same user, high inference accuracy was maintained because of the intelligent compensation algorithm. In this experiment, the signal-to-noise ratio (SNR) of the developed SSI was calculated to be a maximum of 33.75 dB, which, when compared to the typical SNR of 10.17 dB [39] for commercial EMG systems, demonstrates that the SSI developed in this study captures signals much more clearly. This demonstrates the structural robustness of the CVOS-based SSI, as well as the high robustness of the proposed algorithm.

The generalization performance of the developed model was evaluated on a new user (Table S3 and Fig. 4F). To facilitate efficient fine-tuning with limited data from new users, we integrated low-rank adaptation (LoRA) into the Transformer encoder of our SSI. LoRA introduces trainable low-rank matrices while keeping the pretrained model weights frozen, drastically reducing both memory and computational overhead. This approach enables rapid adaptation of the system to new users with minimal data, addressing the challenges posed by interuser variability in silent speech patterns. The LoRA technique, optimized for Transformer models, outperforms the conventional fully connected layer fine-tuning method, achieving an accuracy of 80% compared to 76% with 20 samples per class from new users (Fig. 4G). These results underscore the importance of applying LoRA-based fine-tuning in real-world scenarios to ensure generalizability across diverse users. Additionally, they highlight the potential for further enhancing model performance through continuous data collection integrated with automated learning processes.

### System validation in various physics conditions: Attachment tightness, vocal effort, and body movements

**Fig. 5.**
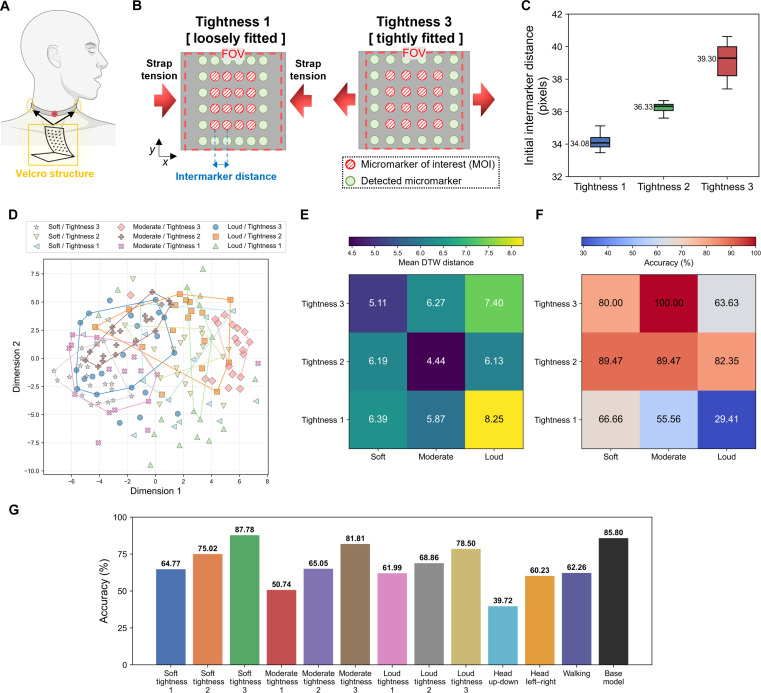
Quantitative evaluation of SSI performance under varying wearing tightness, vocal intensity, and motion artifact conditions. (A) Schematic illustration of the CVOS-based SSI mounted on the neck using an integrated Velcro structure. (B) Conceptual representation of wearing tightness condition detected by the CVOS sensor. (C) Quantification of the initial intermarker distance across tightness levels (Tightness 1, 2, and 3). (D) Two-dimensional latent space visualizations acquired under combinations of vocal intensity (soft, moderate, and loud) and wearing tightness conditions. (E) Heatmap of the mean DTW distance across tightness-vocal intensity. (F) Decoding accuracies visualized as a heatmap. (G) Overall decoding accuracy across varying wearing tightness, vocal intensity, and motion artifact conditions.

The integration of multiaxial strain sensing in the proposed CVOS sensor with an AI-driven signal-processing framework enables highly reliable operation across diverse real-world conditions. The proposed CVOS sensor-based SSI is designed as a detachable and reusable system, incorporating a Velcro-based adjustment mechanism that allows users to independently set the strap tightness (Fig. 5A). In contrast to conventional SSI systems, our multiaxial strain mapping platform quantifies strap tension by measuring the initial intermarker distance (Fig. 5B). Increased tightness shifts the tension outward, reducing both the number of markers captured within the camera’s FOV and the initial intermarker distance. Looser attachment produces the opposite effect, increasing both the marker count and spacing within the FOV. Using these features, strap tightness was classified into 3 levels (Tightness 1 to 3, with larger values indicating a tighter fit) as summarized in Fig. 5C. Speech intensity varies across users and contexts and introduces systematic differences in muscle-strain patterns. To account for this, vocal effort was categorized into 3 levels (Soft <59 dB, Moderate 59 to 67 dB, and Loud >67 dB). Although standard protocols define 4 levels, precise control of vocal intensity was impractical; therefore, a 3-level scheme was adopted. Combining the 3 tightness and 3 intensity conditions yielded 9 experimental states. We analyzed these states using DTW-based K-means clustering and visualized the embeddings using multidimensional scaling, accompanied by heatmaps of the intergroup DTW distances (Figs. 5D and E and Fig. S10). To highlight system reliability independent of linguistic variation, all analyses were performed using a single representative word (“Mike”). As shown in Fig. 5D, cluster boundaries are well separated across both vocal intensity and tightness. Soft and Moderate-intensity utterances form a compact cluster, whereas Loud utterances exhibit broad dispersion across the embedding space. This divergence reflects the inherently higher variability in throat-muscle deformation during loud phonation. Tightness levels 2 and 3 produce particularly concentrated distributions, corresponding to the design-target attachment range, and indicate that reliable decoding accuracy is maintained when the device is not worn excessively loosely. Intragroup DTW distances (Fig. 5E) further quantify these effects. The Moderate/Tightness 2 condition generates the most coherent patterns, exhibiting the smallest mean DTW distance (4.44). In contrast, the Loud/Tightness 1 condition yields the largest distance (8.25), reflecting high variability and limited capture fidelity. Collectively, these findings show that the system provides highly stable signal acquisition when extreme loudness or insufficient tightness is avoided. Decoding performance across all 9 conditions (Fig. 5F) mirrors the DTW-based analysis. The highest accuracy (100%) occurs for Moderate/Tightness 3, whereas the lowest accuracy (29.41%) is observed in Loud/Tightness 1. Reduced performance under Loud conditions arises from rapid, large-amplitude muscle expansions that exceed the sampling capability of the current hardware. The degradation at Tightness 1 is attributed to insufficient skin–device coupling. Nevertheless, Loud/Tightness 2 condition demonstrates that, when the strap is adequately coupled to the neck, even rapid and abrupt strain changes can be captured robustly, suggesting that hardware enhancements such as higher sampling rates could further improve performance. Decoding accuracy across all words (Fig. 5G) exhibits similar trends.

Maintaining intimate mechanical contact with the neck surface, the SSI is susceptible to motion-induced muscle activity that may introduce noise. Maintaining intimate mechanical contact with the neck surface, the SSI is susceptible to motion-induced muscle activity that may introduce noise. Although the study of motion artifacts is essential for the practical deployment of SSI systems, prior work has largely evaluated performance only under static postures. To address this limitation, we assessed decoding accuracy under 3 representative motion conditions: speaking while shaking the head up–down, shaking left–right, and speaking while walking (Fig. 5G). Across all motion cases, substantial degradation in decoding accuracy was observed relative to the baseline model, with the most pronounced reduction occurring during the head up–down condition, yielding a marked decoding accuracy drop to 39.72%. This degradation arises because the baseline data used to train the base model were collected while participants were seated in a relaxed posture, whereas the motion tasks introduce increased muscular tension and altered neck biomechanics, both of which adversely affect the consistency of the strain patterns used for decoding. Because the CVOS sensor captures only multiaxial strain on the throat surface, motion artifacts cannot be fully eliminated using the CVOS sensor alone. These artifacts may be mitigated through sensor-fusion approaches, such as integrating an inertial measurement unit to record underlying inertial dynamics and compensate for body movement-induced perturbations.

### Experimental demonstration in real-world scenarios

The high mechanical stability, exceptional multiaxial strain sensing capabilities, and robustness to external noise of the proposed CVOS sensor support its implementation in next-generation wearable SSI devices designed for practical applications. To demonstrate these advantages, several experiments were conducted to evaluate the overall performance of speech recognition using the CVOS-based SSI in environments involving noise and mechanical vibrations.

**Fig. 6.**
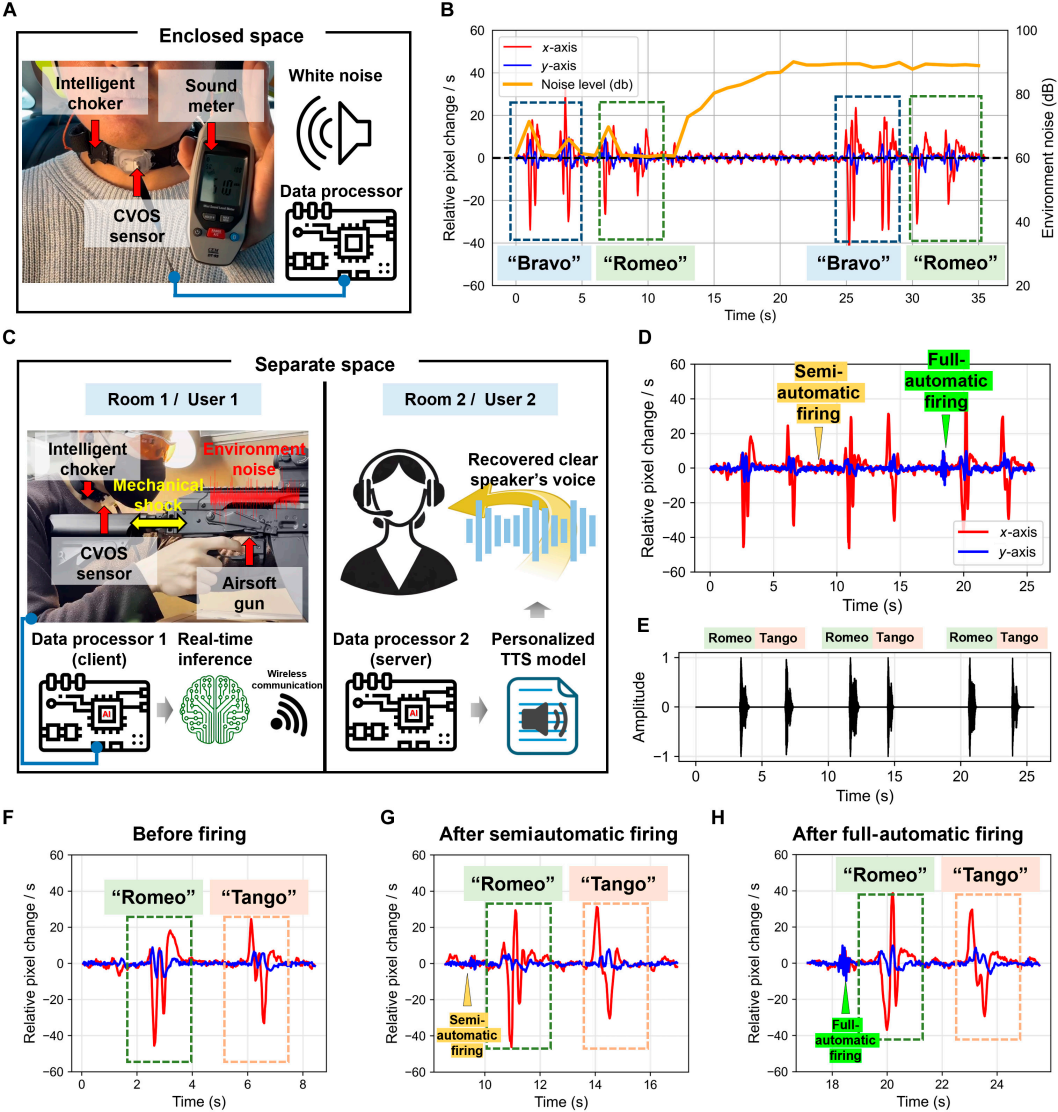
Speech recognition performance of the wearable SSI system combined with the CVOS sensor in the presence of environmental noise in an enclosed space and irregular noise combined with direct physical vibrations. (A) Experimental environment for evaluating word recognition performance in the presence of environmental noise. (B) Real-time inference results of the CVOS-based SSI in the presence of environmental noise. (C) Experimental environment involving firing a rifle for evaluating word-recognition performance in the presence of irregular noise and direct mechanical vibrations. (D) Complete experimental data. (E) Waveforms of synthesized voice. (F) Word-recognition performance before firing. (G) Word-recognition performance after semiautomatic firing. (H) Word-recognition performance after full-automatic firing.

First, the recognition performance was evaluated in the presence of excessive external noise. To this end, a user equipped with the CVOS-based SSI was instructed to pronounce NATO phonetic alphabet words in an environment with 90-dB white noise (Fig. 6A and Movie S5), which is equivalent to noise at a construction site. The speech recognition performance of the CVOS-based SSI was observed to remain the same as the model inference results in a typical 60-dB noise environment (Fig. 6B). Given that interpersonal communication becomes severely difficult at noise levels exceeding 80 dB, comparable to the sound of a motorcycle or vacuum cleaner, the robust performance of the sensor in such high-noise environments indicates its suitability for real-world applications. Furthermore, as shown in Fig. 6A, when the subject held their arm up for a prolonged period, slight vibrations were transmitted as noise to the system, resulting in a lower SNR of 23.18 dB. Despite this, the developed SSI still provides sufficient signal clarity when compared to previous studies (up to 20.87 dB) [18,40]. This highlights the robustness of the CVOS-based SSI, demonstrating its ability to function effectively even with variations in the movement of the human body.

Next, the performance of the SSI system was evaluated in more complex, real-world conditions involving irregular noise and direct physical vibrations in a separate space (Rooms 1 and 2). To this end, User 1, equipped with the CVOS-based SSI, fired a gas blowback rifle in semi- and full-automatic modes in Room 1. The real-time inference results of data processor 1 (client), which is equipped with an AI-based real-time signal processing algorithm, were transmitted to User 2 via wireless communication in Room 2. Data processor 2 (server) recovered the voice signal using the personalized TTS and visualized the words (Fig. 6C). The developed SSI was observed to be capable of distinguishing words corresponding to before and after the firing modes; it was confirmed that the distinguished words could be synthesized into speech and transmitted to another user in real time (Fig. 6D and Movie S6). The waveforms of the synthesized voice were observed to be highly similar to the actual pronunciation of the user (Fig. 6E). Notably, the signal processing algorithm of the developed SSI did not respond to the signal patterns during semiautomatic and full-automatic firing but was observed to react sensitively only to signal patterns corresponding to intended words. Furthermore, minimal changes were observed in the recognition patterns for the same words before and after firing (Figs. 6F to H). This demonstrates the stable operation of the proposed system even in environments involving irregular and intense vibrations, such as battlefields or construction sites. These findings provide important insights into sensor design and algorithm development for next-generation SSI systems.

## Discussion

The CVOS-based SSI integrates a highly reliable CVOS sensor with state-of-the-art AI technologies. This combination enables robust operation and practical applicability in real-world environments. The core component of the CVOS sensor consists of a polymer-based sensing substrate embedded with micro-sized image markers, coupled with a commercially available optical system for tracking their movement. By capturing positional shifts of these micromarkers during substrate deformation, the optical system acquires high-resolution data on both the magnitude and direction of strain at the attachment site. Computer vision-based strain sensing endows the CVOS sensor with a semipermanent operational lifespan, exceptional sensing fidelity, and multiaxial strain map detection capabilities, providing highly reliable data that enhance the inference performance of the AI inference model. These qualities make CVOS sensors a viable alternative to conventional wearable strain sensors based on conductive layers, effectively mitigating their inherent limitations. Furthermore, the performance of the CVOS sensor can be modulated by optimizing the focal length and the fabrication parameters of the micromarkers. In this study, we optimized its capability to capture small, sensitive, and localized deformations of throat muscles (*ε* = 0% to 5%; gauge factor = 3,625). Experimental evaluations conducted in high-noise and high-vibration environments challenge the prevailing notion that vision-based systems are inherently bulky and unsuitable for wearable applications. Contrary to this perception, the developed miniaturized vision system exhibited seamless integration with the soft substrate and conformed closely to the user’s skin, underscoring its potential for practical wearable implementations. Nonetheless, residual concerns regarding the form factor of vision systems can be mitigated through advancements in camera miniaturization, such as lensless camera technology [41–43] and compact microlens camera technology [44–46].

From a practical viewpoint, SSI technologies have evolved as sensing modalities and device form factors have shifted, with these transitions driving continuous improvements in wearability, reusability, signal stability, and decoding accuracy (see Table 1). EEG-SSI extracts neural activity attenuated through the skull, inherently yielding very low SNR (~2 dB [47]) and correspondingly limited decoding accuracy [16,48], though it remains meaningful for fully paralyzed patients [49]. sEMG-SSI is one of the most widely explored modalities, offering moderate SNR (1.5 to 20.8 dB [18,40]) and high decoding accuracy [18,40]; however, it requires conductive gel and skin preparation, substantially reducing wearability and reusability. Early strain sensor-based SSI systems relied on skin-attached patches, which exhibited high SNR (9.5 to 27.1 dB [50]) but suffered from baseline drift [31,51,52] and the need for adhesives, limiting both long-term fidelity and reusability. Despite these limitations, the high decoding accuracy achieved by skin-attached SSI systems has established an important benchmark within the SSI field. In settings where trained personnel are available, such as clinical environments, these systems can serve as a highly practical and effective solution. More recently, platform-attached strain-SSI approaches (e.g., chokers [24] and masks [23]) have improved wearability by eliminating adhesives; however, when normalized by vocabulary size, their decoding accuracy remains lower than that of direct skin-attached systems [9,53] due to reduced signal amplitude and stability. Their limited long-term fidelity continues to impede full transition into practical, real-world use. The proposed CVOS-based SSI advances the field by achieving the highest signal stability reported to date for SSI systems (34 dB) and by providing high-fidelity sensing through nonresistive computer vision-based optical measurements. The system also demonstrates consistently strong decoding performance, achieving an accuracy of 85.8% across 26 words. This performance is competitive with SSIs that encode signals derived from throat-muscle activity, although it remains somewhat lower than that of the highest-performing skin-attached SSI reported in the literature (Fig. 4C). From a practical standpoint, the system offers substantial advantages, including adhesive-free operation, exceptional reusability, and ease of reattachment, all of which contribute to superior real-world usability. From an algorithmic perspective, most recently developed SSI systems, regardless of sensing modality, rely on AI-based classifiers, including GRU (gated recurrent unit), LSTM (long short-term memory), RNN (recurrent neural network), DBN (deep belief network), and CNN architectures, to decode acquired biosignals into linguistic units. Among these approaches, CNN-based classifiers have been most widely adopted due to their strong capability in extracting local spatial features from structured signal representations. However, CNN architectures were originally designed for image processing tasks and therefore tend to emphasize localized features, which limits their ability to capture long-range temporal dependencies and global coarticulatory dynamics that are intrinsic to silent speech signals. While CNN–LSTM hybrid architectures have been investigated, their inherently sequential recurrence and limited ability to perform content-adaptive global temporal reweighting still constrain their capacity to capture complex linguistic dependencies. To address this challenge, the proposed CVOS-based SSI integrates a hybrid CNN–Transformer architecture that combines complementary modeling strengths. Specifically, the CNN component is employed to capture fine-grained spatial deformation cues in multiaxial throat strain maps, while the Transformer module performs temporal reweighting and global alignment across deformation trajectories. As a result, the hybrid model more effectively represents both local and global characteristics of silent speech articulation. Quantitatively, the proposed hybrid architecture achieves a decoding accuracy of 85.8%, outperforming the CNN-only model (81.8%) on our dataset, as shown in Fig. S8. In addition to architectural considerations, intersubject variability in biosignal patterns is a well-recognized challenge in SSI systems. Despite this, prior SSI studies have rarely explored explicit classifier adaptation or personalization strategies. In contrast, the proposed CVOS-based SSI incorporates parameter-efficient model adaptation using LoRA, enabling efficient user-specific personalization modeling with minimal additional data. This approach effectively addresses user-specific variability, which is inherent to wearable SSI. Collectively, these algorithmic features, which incorporate a CNN–Transformer architecture for spatiotemporal modeling of speech-related strain signals and LoRA-driven efficient personalization, further distinguish the proposed CVOS-based SSI as a practically deployable and scalable solution compared to existing SSI approaches. When considered together with the inherently high SNR and signal reliability provided by the CVOS sensor, these practical and algorithmic attributes highlight the scalability of the platform and its potential to evolve into an increasingly practical and high-performance SSI solution.

**Table 1.** Comparative analysis of signal stability, wearability, reusability, sensor, decoding accuracy, and algorithms in SSI systems

Metric	EEG-SSI	sEMG-SSI	Skin-attached strain-SSI	Platform-attached strain-SSI	CVOS-based SSI
Signal stability (SNR)	Low (2 dB [47])	Moderate (1.5–20.8 dB [18,40])	High (9.5–27.1 dB/in-room condition [50])		Very high (34 dB)
Decoding accuracy	80% [48] (5 words)	77.6% [40] (5 words)	80% [53] (8 words)	84.4% [23] (21 words)	85.8% (26 words)
	85% [16] (9 words)	87.5% [18] (100 words)	92.6% [9] (110 words)	95.2% [24] (20 words)	
Wearability	Very low (electrode headgear)	Low (skin patch)	Low (skin patch)	High (choker/mask)	High (choker)
Reusability	Very low (fixed setup)	Very low (gel drying)	Low (gel drying, low fidelity)	High (gel-free, low fidelity)	Very high (gel-free, high fidelity)
Sensor attachment	Skin	Skin	Skin	Platform	Platform
Sensing mechanism	Electric	Electric	Electric	Electric	Computer vision
Classifier architecture	GRU [16], RNN [48], DBN [48]	CNN [18], LDA [40]	CNN [9]	CNN [23,24]	CNN–Transformer
Classifier adaptation approach	–	–	–	Fine-tuning [24]	LoRA

In practical scenarios, variations in wearable SSI attachment inevitably introduce mechanical baseline differences that persist across repeated usage. The initial residual stress map provides a physically interpretable representation of the preexisting geometric state of the sensor–skin interface prior to intentional speech-related muscle activation. Rather than reflecting dynamic vocal motion, this map captures the initial spatial distribution of attachment-induced deformation arising from factors such as strap tightness, effective sensor–skin conformity, and inter- and intrasubject variability in sensor placement. This concept is inspired by the notion of residual stress in solid mechanics, which describes stresses that remain within a material after the original external causes have been removed. Analogously, even in the absence of active phonation, the wearable SSI retains a nonzero mechanical baseline that influences subsequent multiaxial strain mapping. By explicitly modeling this baseline as the initial residual stress map, the proposed framework introduces a physically grounded reference state that enables systematic interpretation of trial-to-trial variations in strain patterns. From a signal processing perspective, incorporating the initial residual stress map into the adaptive inference pipeline allows the AI model to decouple static attachment-induced signals from speech-induced dynamic strain signals. This separation enhances inference robustness under repeated reattachment and across users, addressing a key limitation of prior data-driven SSI approaches that implicitly absorb such variability into model parameters without explicit physical interpretation. By leveraging this map to infer the attachment state, the AI model can automatically perform subject- and trial-specific baseline calibration, without requiring additional manual adjustment. Consequently, the proposed approach bridges solid-mechanics-inspired physical reasoning with AI-based inference, contributing to both improved generalization performance and enhanced interpretability of the SSI model.

To prioritize clear and efficient communication using a limited set of words, the CVOS-based SSI focuses on recognizing the 26-word NATO phonetic alphabet. This is because SSI systems that accurately recognize human speech at the sentence level are not aligned with the primary use case of SSI, which is intended for noisy environments, and face clear limitations with regard to implementations using current technology. In contrast, a system tailored for NATO phonetic alphabet recognition, which relies on a constrained vocabulary set validated for high-noise conditions, enables immediate application in various industrial and operational settings. While NATO phonetic-based communication is inherently more accessible to trained users, the integration of TTS technology facilitates message reconstruction, enabling nonexpert listeners to interpret spoken phonetic sequences as fully synthesized sentences.

The primary contribution of this study is the integration of adaptive AI-driven methodologies into the SSI framework. These methods explicitly account for sensor alignment variations, external environmental conditions, and individual biomechanical differences. As a result, both system usability and inference reliability are considerably improved. The proposed framework employs a high-performance AI inference model to analyze the multiaxial strain map alongside a personalized TTS model that reconstructs spoken output in the speaker’s synthesized voice, substantially enhancing user experience. The AI inference model is intricately and meticulously structured to comprehend the global context of captured patterns during the real-time process. A signal compensation algorithm in the AI inference model, designed to maintain sensor alignment, ensures consistent inference results across diverse signal patterns obtained from over 100 reattachments spanning several months. The performance of the AI inference model can be continuously improved through future integration with an automated learning algorithm, which will collect and train on new data each time the SSI is used. Furthermore, the AI models can be fine-tuned using minimal data via transfer learning, demonstrating adaptability to individual vocalization styles, device placement variations, and user-specific physiological factors. By comprehensively understanding the multiaxial strain map captured by the throat-mounted CVOS sensor, this SSI system paves the way for next-generation wearable technologies.

To enable practical SSI applications that require both wearability and portability, the proposed system was designed to fully transition toward edge computing. Except for offline AI model development, all experimental demonstrations were performed on an embedded platform (Raspberry Pi 5) equipped with an integrated tiny camera module and the proposed software framework. To support real-time on-device inference, the AI architecture was lightweighted using a knowledge distillation (Teacher–Student) approach, reducing the model size from 12.4 to 3.6 MB and improving inference latency from 0.018 to 0.003 s. The optimized on-board software enabled real-time AI-based signal processing at the edge, and the computational margin gained through model compression further allowed real-time TTS synthesis to run concurrently. The physical form factor of the hardware can be further minimized in accordance with advances in embedded computing performance and device miniaturization, and the computational load of the software may also be reduced through future AI compression techniques. This hardware–software co-optimization grounded in edge computing ensures the low-latency, high-reliability processing required for practical SSI operation, ultimately enabling the system to function as a realistic communication tool even in high-noise environments.

Despite these advancements, we acknowledge that the sequential execution of high-performance AI models and the image processing latency of the CVOS sensor constrain the overall system processing speed (sampling rate = 50 Hz). However, given that this rate aligns with the biological frequency range of muscle signals (<60 Hz [54,55]), it remains sufficient for robust strain-based speech inference, as demonstrated by the model’s predictive performance. Nonetheless, to further optimize real-time processing capabilities, future research will focus on transitioning the AI architecture to an end-to-end framework, refining image processing methodologies, and leveraging AI acceleration hardware to enhance signal processing throughput. By seamlessly integrating multiaxial strain sensing with cutting-edge AI technologies into a reusable and highly reliable interface, the proposed system represents a pivotal step toward enabling effective communication in acoustically challenging environments.

Future work will focus on advancing the proposed CVOS-based SSI from a foundational feasibility demonstration toward a fully scalable silent speech communication platform. While the present study prioritized establishing a reliable sensing and modeling framework under controlled conditions, subsequent research will expand dataset size and diversity by incorporating a larger participant cohort, multisession recordings, and broader lexical sets, thereby enabling rigorous model validation and supporting the transition to large vocabulary and eventually continuous silent speech decoding. Practical deployment further necessitates deeper characterization of motion artifacts; thus, additional experiments involving complex movement scenarios, systematic assessment of posture-dependent muscular tension, and integration of inertial measurement unit-based inertial sensing will be conducted to enhance robustness under real-world conditions. In parallel, engineering improvements will aim to optimize the wearable platform through more ergonomic attachment structures, repeatable reattachment protocols, and long-term durability testing, thereby strengthening reusability and user comfort. Collectively, these research directions will enable the CVOS-SSI to evolve into a motion-robust, large-vocabulary, and user-friendly system suitable for practical deployment across clinical, industrial, and everyday communication environments.

## Data Availability

The code of real-time AI processing is on our GitHub, and the URL is https://github.com/HongSungUk/CVOS-sensor-block2. All data are available within the article or available from the authors upon reasonable request.
