# Supplementary material for: Soft Multiaxial Strain Mapping Interface with AI-Driven Decoding for Silent Speech in Noise
Source: Cyborg Bionic Syst. 2026 Mar 23;7:0536. doi: 10.34133/cbsystems.0536 (PMC13006734; doi:10.34133/cbsystems.0536)
Supplement: Supplementary 1 — Figs. S1 to S10 Tables S1 to S8 Movies S1 to S6 [file cbsystems.0536.f1.zip › 260120_supplementary_materials_v3.docx]

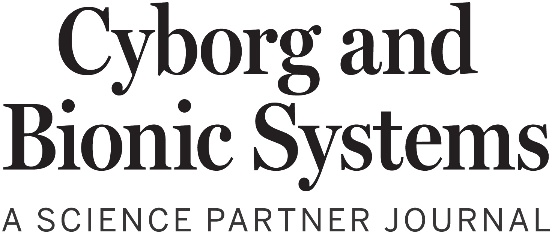

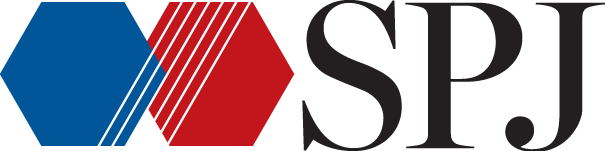


Supplementary Materials for

**Soft multiaxial strain mapping interface with AI-driven decoding for silent speech in noise**

Sunguk Hong *et al.*

*Corresponding author. Email: sungminpark@postech.ac.kr

**This PDF file includes:**

Figs. S1 to S10

Tables S1 to S8

Legends for movies S1 to S6

**Other Supplementary Materials for this manuscript include the following:**

Movies S1 to S6

Supplementary Figures

**
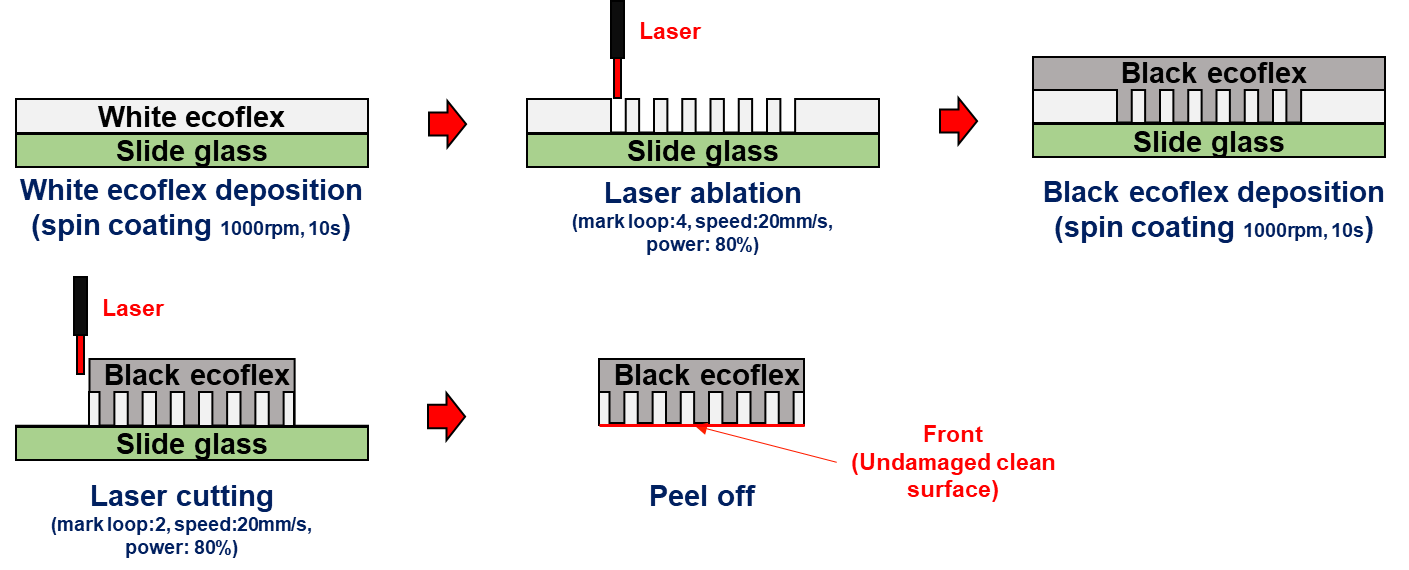
**

**Fig. S1. Fabrication process of the CVOS sensor’s sensing part.**

**
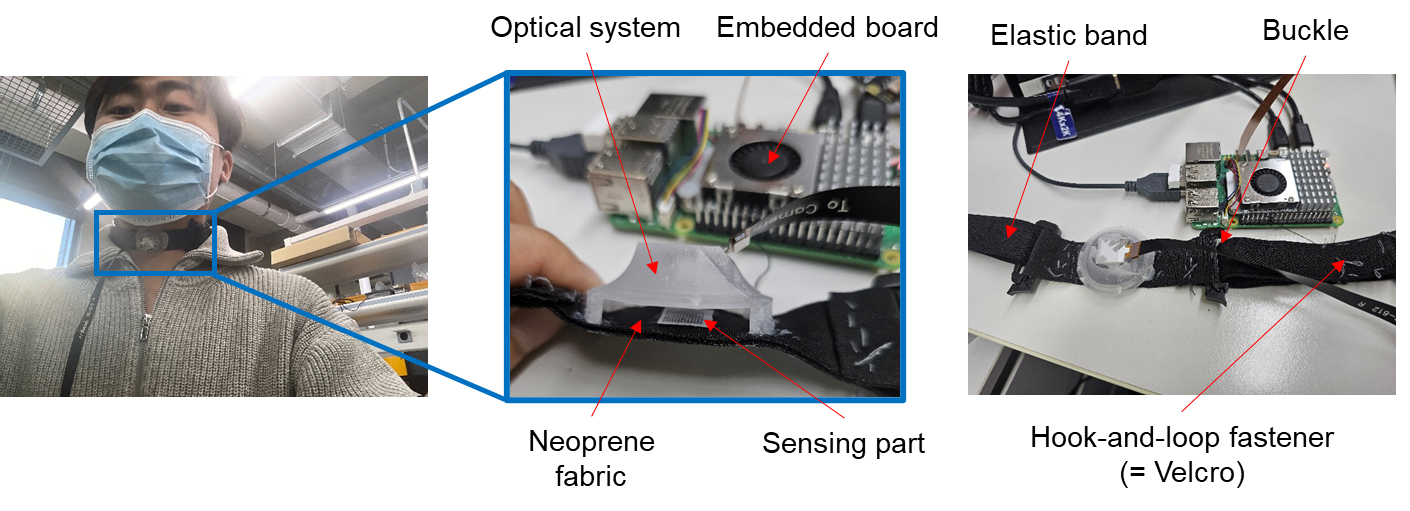
 Fig. S2. Neck chocker combined with the CVOS sensor.**

**
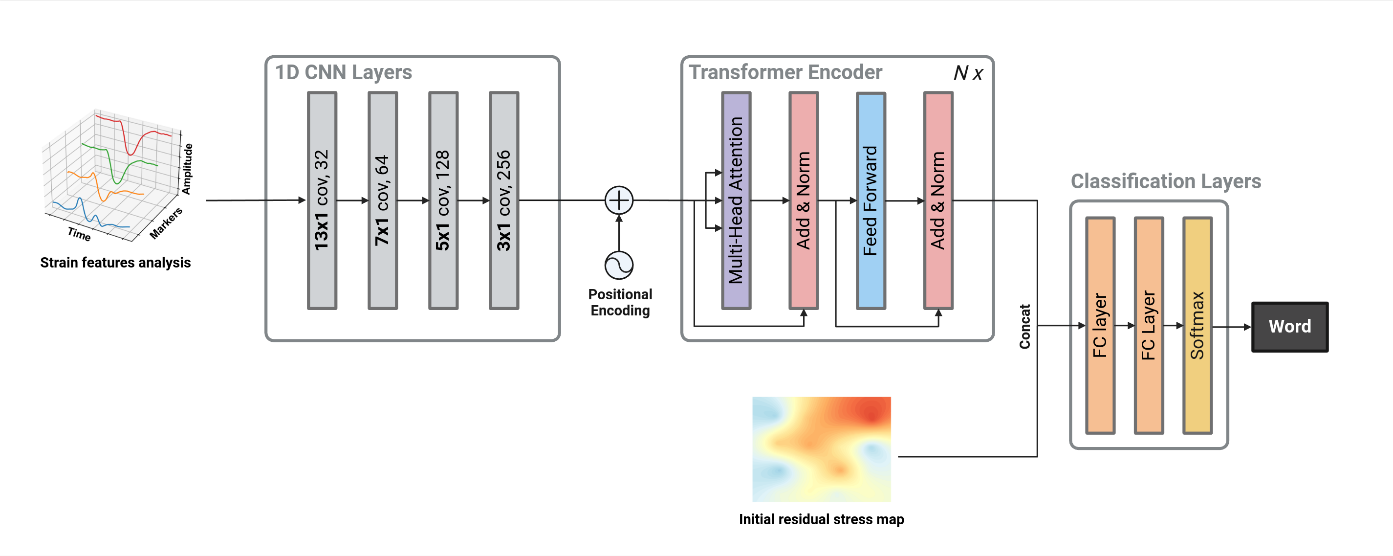
Fig. S3. Details of the model backbone.**


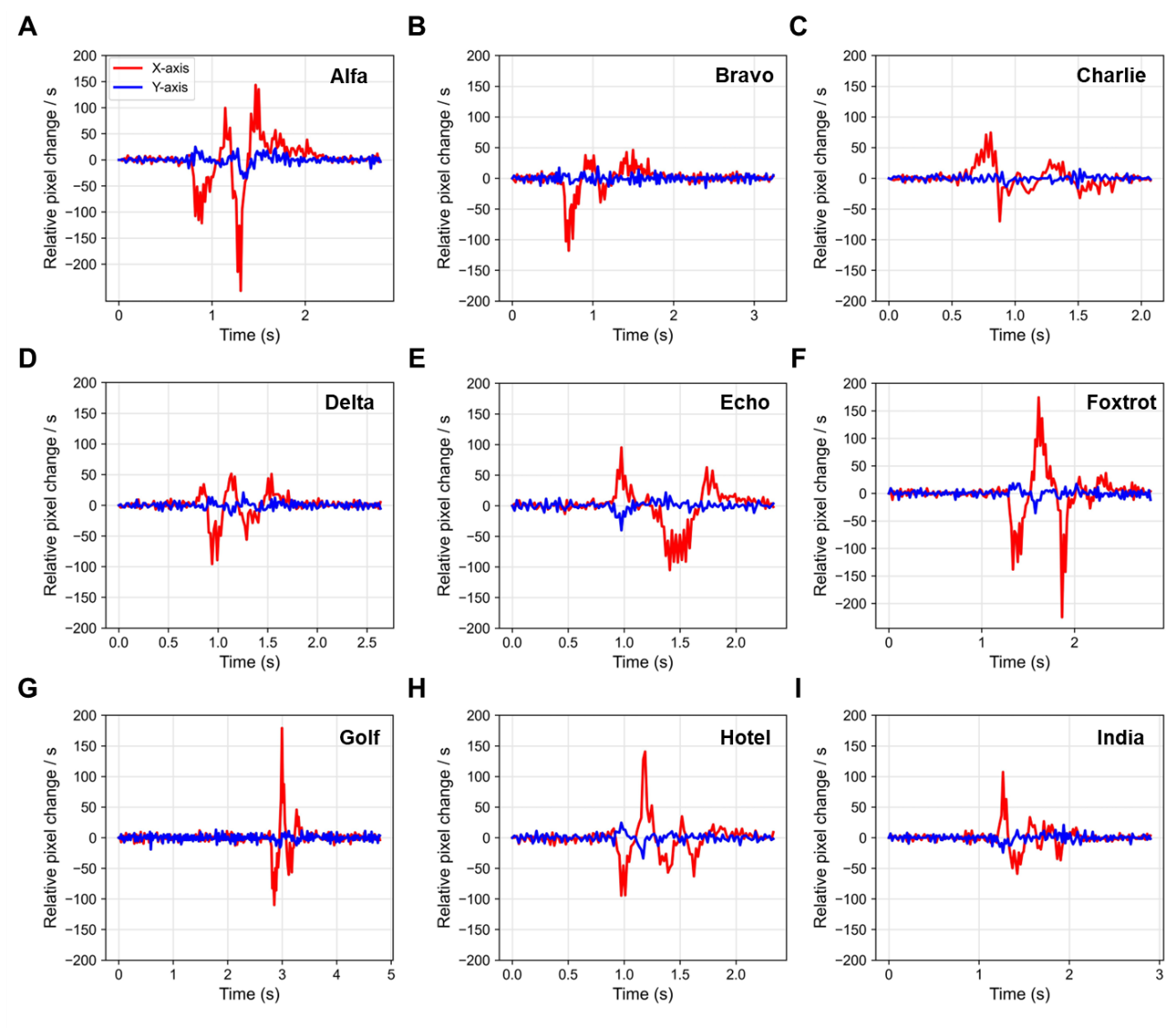
**Fig. S4. Relative pixel change per second of the NATO phonetic alphabets: Alfa - India.** (**A**) Alfa, (**B**) Bravo, (**C**) Charlie, (**D**) Delta, (**E**) Echo, (**F**) Foxtrot, (**G**) Golf, (**H**) Hotel, and (**I**) India.

**
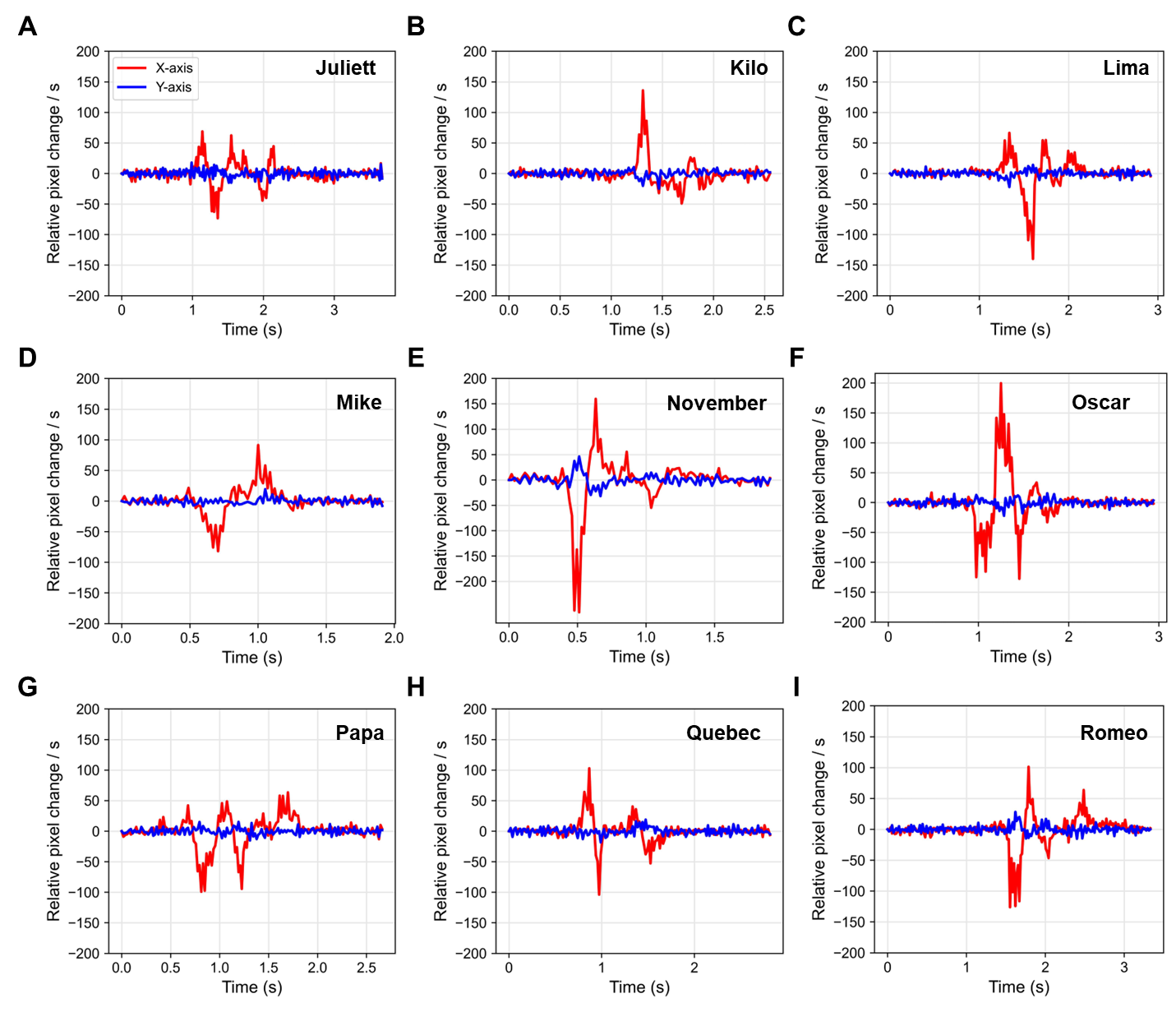
**

**Fig. S5. Relative pixel change per second of the NATO phonetic alphabets: Juliett - Romeo.** (**A**) Juliett, (**B**) Kilo, (**C**) Lima, (**D**) Mike, (**E**) November, (**F**) Oscar, (**G**) Papa, (**H**) Quebec, and (**I**) Romeo.

**
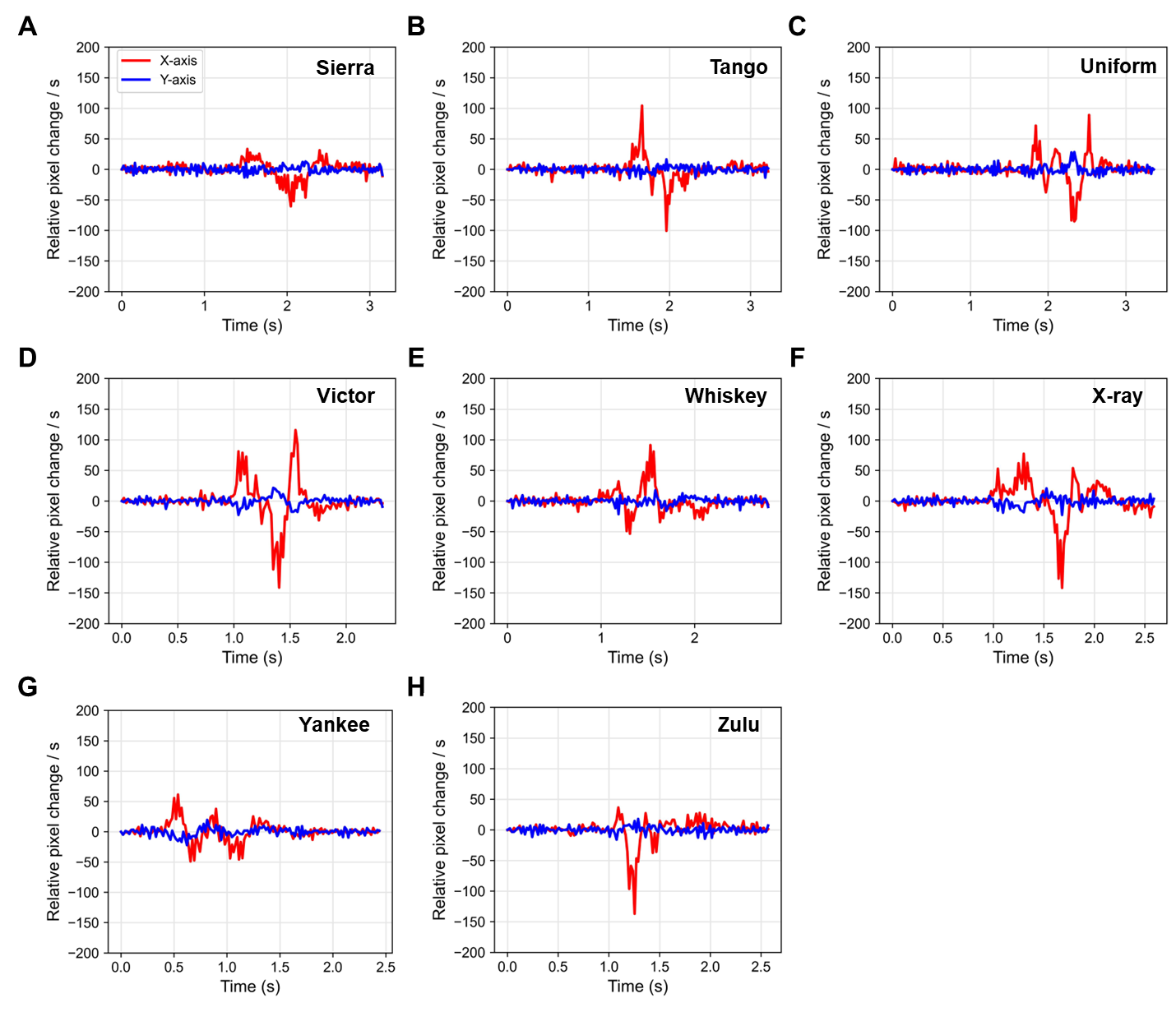
**

**Fig. S6. Relative pixel change per second of the NATO phonetic alphabets: Sierra - Zulu.**

(**A**) Sierra, (**B**) Tango, (**C**) Uniform, (**D**) Victor, (**E**) Whiskey, (**F**) X-ray, (**G**) Yankee, and (**H**) Zulu.

**
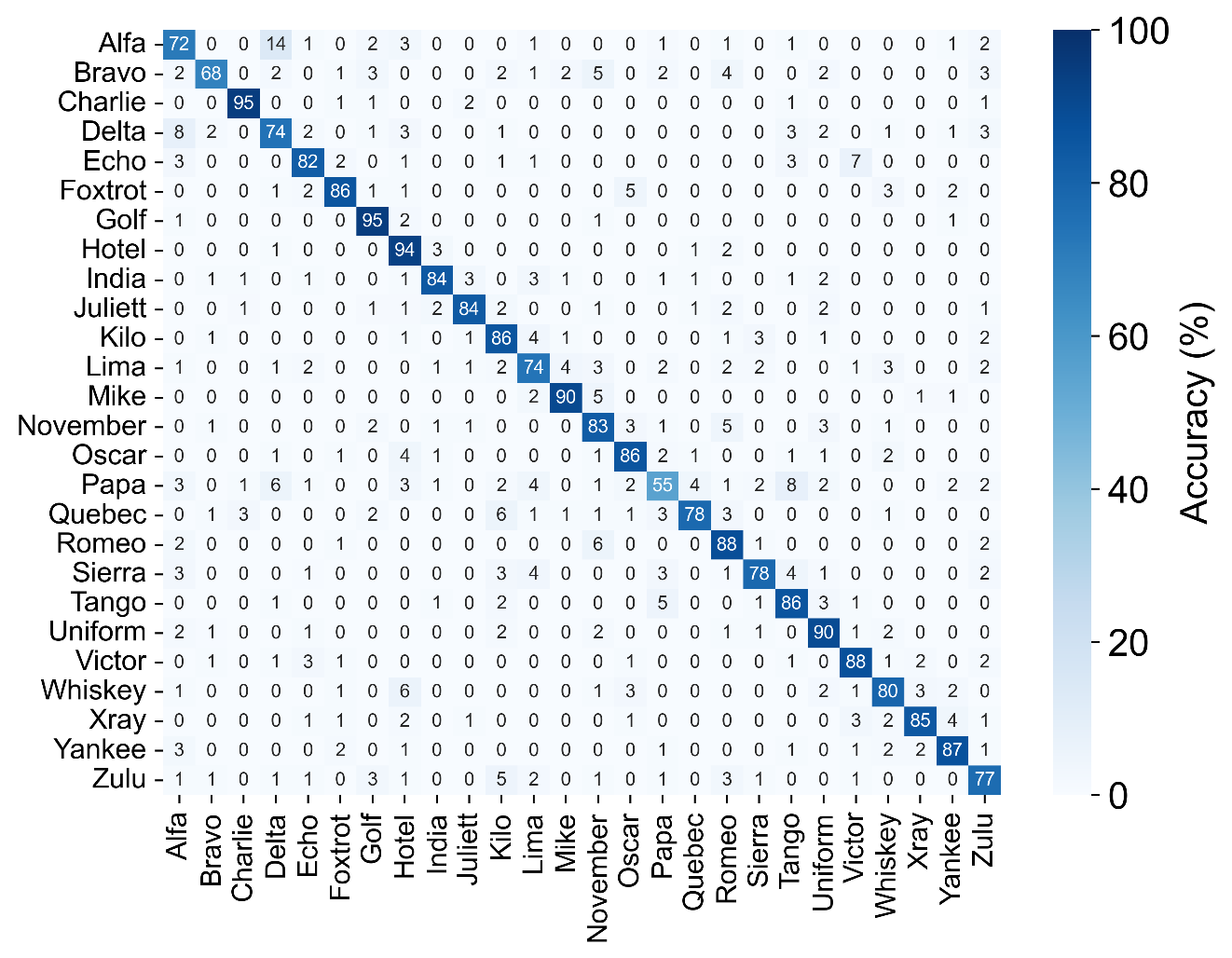
**

**Fig. S7. Confusion matrix showing the classification results.**

**
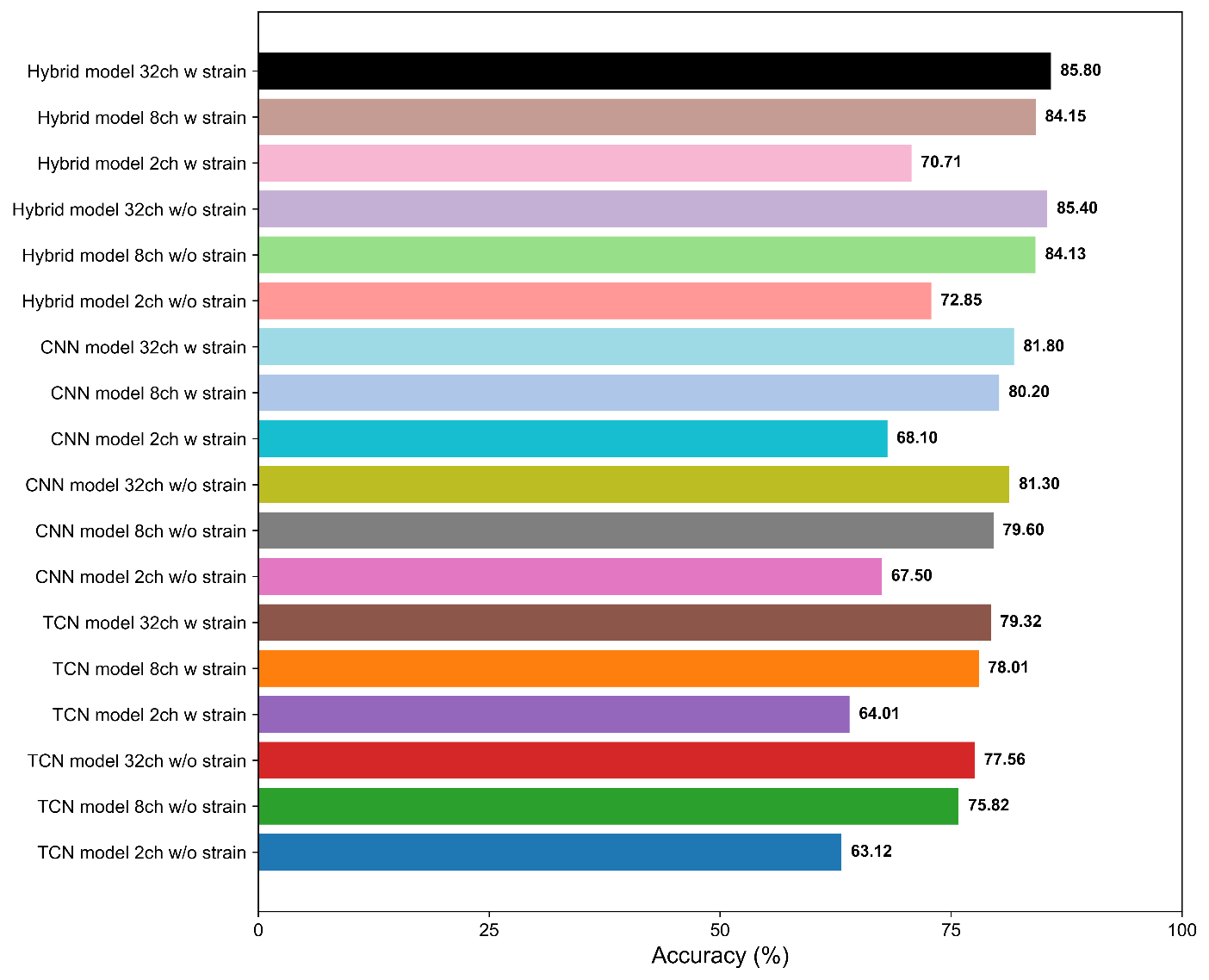
Fig. S8. Overall decoding accuracy with channel and strain map: TCN model, CNN model, and Hybrid (CNN-Transformer) model.**

**
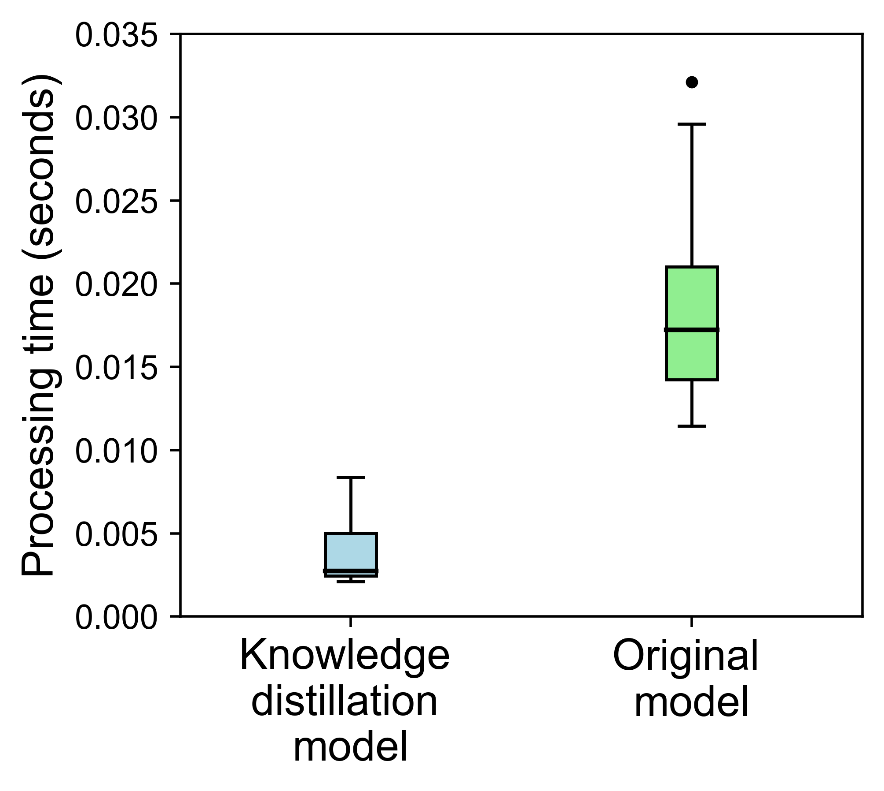
**

**Fig. S9. Comparison of processing time: knowledge distillation model vs original model.
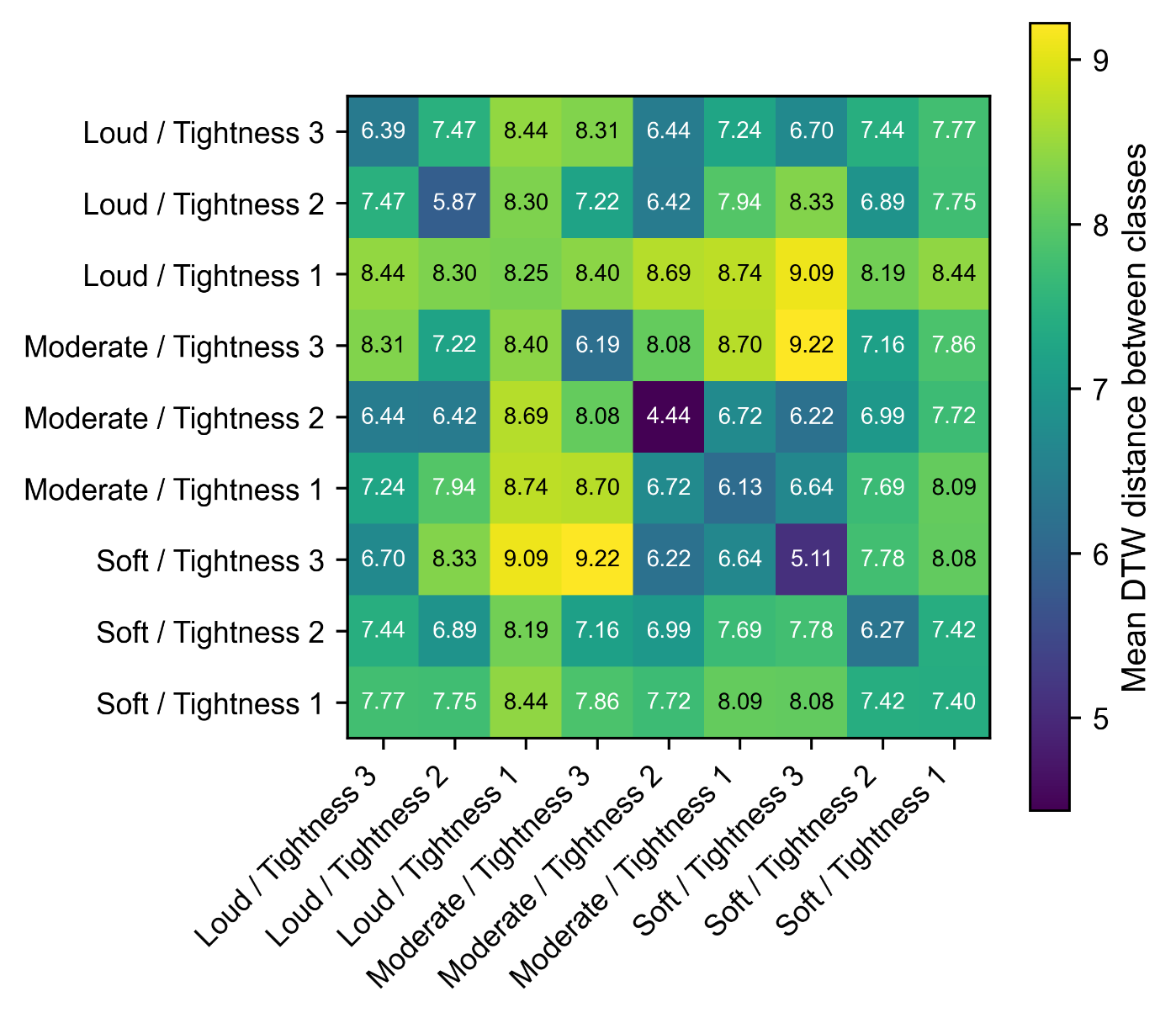
**

**Fig. S10. Heatmap of the inter or in-group mean DTW distances.**

Supplementary Tables

| **Module** | **Component** | **Parameter / Property** | **Teacher Model** | **Student Model** |
| --- | --- | --- | --- | --- |
| Input | Data | Input Channels | [2 / 8 / 32] | [2 / 8 / 32] |
| 1D CNN Layers | Conv Block 1 | Kernel / Stride / Pad | 13/1/0 | 13/1/0 |
|  |  | In → Out Channels | [2 / 8 / 32]→32 | [2 / 8 / 32]→16 |
|  | Conv Block 2 | Kernel / Stride / Pad | 7/1/0 | 7/1/0 |
|  |  | In → Out Channels | 32→64 | 16→32 |
|  | Conv Block 3 | Kernel / Stride / Pad | 5/1/0 | 5/1/0 |
|  |  | In → Out Channels | 64→128 | 32→64 |
|  | Conv Block 4 | Kernel / Stride / Pad | 3/1/0 | 3/1/0 |
|  |  | In → Out Channels | 128→256 | 64→128 |
| Transformer  Encoder | Transformer | Dim (dmodel​ / dff​) | 256/1024 | 128/512 |
|  |  | Heads / Layers | 4-2 | 2-1 |
| Classifier Layers | Classifier | Linear 1 (In → Out) | 272→128 | 144→64 |
|  |  | Linear 2 (Out) | *N_classes_​* | *N_classes_​* |

* A 16-dimensional initial residual stress map is incorporated between ‘the Transformer Encoder’ and ‘the Classifier Layers’.

**Table S1. Structural comparison of the teacher and student network.**

|  | **Optimal hyperparameters** |
| --- | --- |
| Optimizer | Adam |
| Learning rate | 3e-4 |
| Dropout rate | 0.1 |
| Epochs | 3000 |
| Batch size | 256 |

**Table S2. Hyperparameters for the classifier.**

| **Number of participants** | **SSI**  **experience** | **Gender** | **Voice-related diseases** | **Age** | **Native language** |
| --- | --- | --- | --- | --- | --- |
| 1 | Old user | Male | None | 32 | Korean |
| 2 | Old user | Male | None | 26 | Korean |
| 3 | Old user | Female | None | 24 | Korean |
| 4 | Old user | Female | None | 25 | Korean |
| 5 | Old user | Male | None | 25 | Korean |
| 6 | Old user | Male | None | 23 | Korean |
| 7 | New user | Female | None | 26 | Korean |

**Table S3. Personal information about the participants.**

| **Ref** | **Figure tag** | **Sensing**  **Methods**  **(material)** | **Gauge factor** | **Working range**  **(%)** | **Detection limit**  **(%)** | **Linearity**  **(*R*^2^)** | **Durability (cycles)** |
| --- | --- | --- | --- | --- | --- | --- | --- |
| This work | This work | Computer-vision  (Ecoflex) | 3625 | 5 | 0.02< | 0.999 | 10000 >  (< 2% strain) |
| 62 | A | Piezo-resistive  (AuNP ^a)^- PI ^b)^) | 19.94 | 1 | 0.08 | 0.99 | No information |
| 63 | B | Piezo-resistive  (PANI ^c)^ - Ecoflex) | 74.28 | 1 | 0.3 | 0.99 | No information |
| 25 | C | Piezo-resistive  (Pt ^d)^ – PU ^e)^) | 5169 | 1 | 0.2< | 0.998 | 2000 |
| 24 | D | Piezo-resistive  (Graphene – SDC ^f)^) | 317 | 5 | 0.05 | No information | 10000 > |
| 56 | E | Piezo-resistive  (Au ^g)^ – PDMS ^h)^) | 2557.71 | 15 | 0.1 | No information | 10000  (5% strain) |
| 57 | F | Piezo-resistive  (EGaIn ^i)^ - hydrogel) | 1.61~7.16 | 400 | 0.1 | No information | 300 |
| 58 | G | Piezo-resistive  (AgNP ^a)^/CNT ^j)^ – TPU ^k)^) | 354 | 640 | 0.1 | No information | 1000  (200% strain) |
| 59 | H | Piezo-resistive  (CNT ^j)^ – PET i^)^/PU ^e)^) | 60 | 50 | 0.1 | 0.997 | 10000> |
| 60 | I | Piezo-resistive  (RGO ^m)^/MWCNT ^n)^ – NR ^o)^) | 1451 | 32 | 0.5 | No information | 1000 |
| 61 | J | Piezo-resistive  (CB ^p)^/MWCNT ^o)^/TPU ^k)^ - elastic yarns) | 9 | 15 | 0.2 | No information | 2500  (10% strain) |

1. AuNP: Gold nanoparticle
2. PI: polyimide
3. PANI: polyaniline
4. Pt: platinum
5. PU: polyurethane
6. SDC: sodium deoxycholate
7. Au: Gold
8. PDMS: polydimethylsiloxane
9. EGaIn: eutectic gallium-indium
10. CNT: carbon nanotube
11. TPU: thermoplastic polyurethane
12. PET: polyethylene terephthalate
13. RGO: reduced graphene oxide
14. MWCNT: multiwalled carbon nanotube
15. NR: natural rubber
16. CB: carbon black

**Table S4. Performance comparison of the strain sensors.**

| **Character** | **Telephony** | **Pronunciation** |
| --- | --- | --- |
| A | Alfa | AL-FAH |
| B | Bravo | BRAH-VOH |
| C | Charlie | CHAR-LEE or SHAR-LEE |
| D | Delta | DELL-TAH |
| E | Echo | ECK-OH |
| F | Foxtrot | FOKX-TROT |
| G | Golf | GOLF |
| H | Hotel | HOH-TEL |
| I | India | IN-DEE-AH |
| J | Juliett | JEW-LEE-ETT |
| K | Kilo | KEY-LOH |
| L | Lima | LEE-MAH |
| M | Mike | MIKE |
| N | November | NO-VEM-BER |
| O | Oscar | OSS-CAH |
| P | Papa | PAH-PAH |
| Q | Quebec | KEH-BECK |
| R | Romeo | ROW-ME-OH |
| S | Sierra | SEE-AIR-RAH |
| T | Tango | TANG-GO |
| U | Uniform | YOU-NEE-FORM or OO-NEE-FORM |
| V | Victor | VIK-TAH |
| W | Whiskey | WISS-KEY |
| X | Xray | ECKS-RAY |
| Y | Yankee | YANG-KEY |
| Z | Zulu | ZOO-LOO |

**Table S5. NATO Phonetic code.**

| **Module** | **Component** | **Parameter / Property** | **Channel** |
| --- | --- | --- | --- |
| Input | Data | Input Channels | 32 |
| TCN Layers | Conv Block | Kernel / Stride / Pad | 7 / 2 / 3 |
|  |  | In → Out Channels | 32→32 |
|  | MaxPool1d | Kernel / Stride / Pad | 3 / 2 / 1 |
|  | TCN Block 1 | Dilation | 1 |
|  |  | In → Out Channels | 32→32 |
|  | TCN Block 2 | Dilation | 2 |
|  |  | In → Out Channels | 32→64 |
|  | TCN Block 3 | Dilation | 4 |
|  |  | In → Out Channels | 64→128 |
|  | TCN Block 4 | Dilation | 8 |
|  |  | In → Out Channels | 128→256 |
| Pooling Layer | AvgPool | Output Dim | 256 |
| Classifier Layers | Classifier | Linear (In → Out) | 272→ *N_classes_​* |

* A 16-dimensional initial residual stress map is incorporated between ‘the Pooling Layer’ and ‘the Classifier Layers’.

**Table S6. Structural of TCN model. The model architecture is adapted from prior related work [24].**

| **Module** | **Component** | **Parameter / Property** | **Channel** |
| --- | --- | --- | --- |
| Input | Data | Input Channels | 32 |
| CNN Layers | Conv Block | Kernel / Stride / Pad | 7 / 2 / 3 |
|  |  | In → Out Channels | 32→32 |
|  | MaxPool1d | Kernel / Stride / Pad | 3 / 2 / 1 |
|  | CNN Block 1 | In → Out Channels | 32→32 |
|  |  | Kernel / Stride | 3 / 1 |
|  | CNN Block 2 | In → Out Channels | 32→64 |
|  |  | Kernel / Stride | 3 / 2 |
|  | CNN Block 3 | In → Out Channels | 64→128 |
|  |  | Kernel / Stride | 3 / 2 |
|  | CNN Block 4 | In → Out Channels | 128→256 |
|  |  | Kernel / Stride | 3 / 2 |
| Pooling Layer | AvgPool | Output Dim | 256 |
| Classifier Layers | Classifier | Linear (In → Out) | 272→ *N_classes_​* |

* A 16-dimensional initial residual stress map is incorporated between ‘the Pooling Layer’ and ‘the Classifier Layers’.

**Table S7. Structural of CNN model. The model architecture is ResNET.**

| **Ref** | **Name** | **Sensing position** | **Reusability** | **Number of words** | **Accuracy** |
| --- | --- | --- | --- | --- | --- |
| This work | This work | Throat brace | Good | 26 | 87.86% |
| 56 | Face skin 1 | Face skin | Bad | 5 | 85.24% |
| 18 | Face skin 2 | Face skin | Bad | 100 | 87.53% |
| 19 | Face skin 3 | Face skin | Bad | 110 | 92.64% |
| 53 | Face skin 4 | Face skin | Bad | 8 | 80.00% |
| 23 | Face mask | Face mask | Good | 21 | 84.40% |
| 9 | Throat skin 1 | Throat skin | Bad | 3 | 85.00% |
| 13 | Throat skin 2 | Throat skin | Bad | 5 | 96.00% |
| 24 | Throat brace | Throat brace | Good | 20 | 95.25% |

**Table S8. Performance comparison with state-of-the-art silent speech systems.**

**Supplementary Movies**

**Movie S1.** MOI tracking of the CVOS sensor

**Movie S2.** Baseline measurement of the CVOS sensor with white noise

**Movie S3.** Real-time monitoring of the CVOS sensor

**Movie S4.** Real-time monitoring and AI inference of the silent speech interface with the CVOS sensor

**Movie S5.** Real-time monitoring and AI inference of the silent speech interface with the CVOS sensor in noise environment

**Movie S6.** Real-time monitoring and AI inference of the silent speech interface with the CVOS sensor during airsoft gun firing
